# pxGPT: a schema-constrained multimodal large language model framework for plant phenotyping

**DOI:** 10.1093/bioinformatics/btag781

**Published:** 2026-10-20

**Authors:** Po-Xing Zheng, Jheng-Yan Ou, Yu-Lin Wu, Ruei-Shiuan Wang, Ting-Li Wu, Te-Chang Hsu, Yao-Cheng Lin

**Affiliations:** Biotechnology Center in Southern Taiwan, Academia Sinica, Tainan 711010, Taiwan; Agricultural Biotechnology Research Center, Academia Sinica, Taipei 115201, Taiwan; Biotechnology Center in Southern Taiwan, Academia Sinica, Tainan 711010, Taiwan; Agricultural Biotechnology Research Center, Academia Sinica, Taipei 115201, Taiwan; Biotechnology Center in Southern Taiwan, Academia Sinica, Tainan 711010, Taiwan; Agricultural Biotechnology Research Center, Academia Sinica, Taipei 115201, Taiwan; Biotechnology Center in Southern Taiwan, Academia Sinica, Tainan 711010, Taiwan; Agricultural Biotechnology Research Center, Academia Sinica, Taipei 115201, Taiwan; Biotechnology Center in Southern Taiwan, Academia Sinica, Tainan 711010, Taiwan; Agricultural Biotechnology Research Center, Academia Sinica, Taipei 115201, Taiwan; Biotechnology Center in Southern Taiwan, Academia Sinica, Tainan 711010, Taiwan; Agricultural Biotechnology Research Center, Academia Sinica, Taipei 115201, Taiwan; Biotechnology Center in Southern Taiwan, Academia Sinica, Tainan 711010, Taiwan; Agricultural Biotechnology Research Center, Academia Sinica, Taipei 115201, Taiwan; Biotechnology Center, National Chung Hsing University, Taichung 402202, Taiwan

## Abstract

**Summary:**

Deep learning can scale image-based plant phenotyping but typically targets predefined traits, whereas manual phenotypic description is more flexible but difficult to scale. We present Phenotype eXplorer Generative Plant Traits (pxGPT), a Python framework that converts multi-view plant images into structured phenotype records. The workflow combines free-text description, dataset-specific schema generation with human review, and schema-constrained information extraction. We evaluated the extraction stage on three *Brassica* datasets using 78 973 annotations from 10 annotators who had no access to pxGPT predictions. For 38–45 qualitative traits per dataset, pxGPT-human agreement ranged from 0.572 to 0.712, compared with 0.620 to 0.739 agreement among human annotators. All 32 736 required trait fields were present; 93 values were empty, and no qualitative descriptor violations were recorded. For selected morphological structures, pooled false-positive rates were 0.5%–8.0%, while sensitivity varied across structure types in one mature-plant dataset (37.5%–100%). The curated schemas were applied unchanged with two commercial models and one locally hosted open-weight model, with differences in mean accuracy of 0.03 or less. In the evaluated *Brassica* datasets, these results support schema-guided extraction of visible plant traits without separate model training for each trait. Human-guided schema development offers a path to extend pxGPT to other crops and imaging conditions, with accuracy requiring validation in each new setting.

**Availability and implementation:**

The software is available at https://github.com/YaoChengLab/pxgpt and an archival version of the source code is available on Zenodo (https://doi.org/10.5281/zenodo.22931745). Curated schemas used in this study, annotation guides, phenotype records, and human annotations are available at https://doi.org/10.5281/zenodo.22907554.

## 1 Introduction

High-throughput imaging and deep learning enable plant phenotyping at population scale, yet most implementations quantify a predetermined set of measurements or represent images as latent features of limited interpretability (Murphy *et al.* 2024, Ubbens *et al.* 2025). Visible traits not anticipated during experimental design are consequently seldom recorded. Generative artificial intelligence has been applied to phenotype description, disease detection, and analytical support (Zhao *et al.* 2024, Chen *et al.* 2025, Yang *et al.* 2025), although the outputs are typically unconstrained text. Crop and trait ontologies provide controlled vocabularies for comparability across experiments (Arnaud *et al.* 2020), but their coverage is limited to curated traits.

An alternative approach is to describe visible phenotypes in natural language and to derive the relevant trait structure from those descriptions. Multimodal large language models (LLMs) generate descriptions of this form from images at scale, but free text cannot be queried or compared systematically. Schema-constrained extraction addresses this limitation by converting unstructured inputs into records conforming to a defined output schema (Caufield *et al.* 2024, Dagdelen *et al.* 2024). Existing approaches, however, define the schema in advance, so omitted traits remain unrecorded.

We present Phenotype eXplorer Generative Plant Traits (pxGPT), a three-step procedure for structured phenotype extraction from plant images. A dataset-specific schema is first derived from open-ended image descriptions. The schema is curated by the user and fixed while samples are processed into conforming records. Descriptors absent from existing ontologies are thereby representable without trait-specific training data or per-trait classifiers. We evaluated extraction on three *Brassica* datasets comprising 321 cultivars sampled across multiple growth stages against independent human annotations of 38–45 traits.

## 2 Implementation

pxGPT is a Python toolkit with three steps (Fig. 1). In Step 1, all available views of each sample are provided together to a commercial vision-capable multimodal LLM (Claude Sonnet 5, Anthropic, USA). The model generates one free-text description of the sample’s visible morphology.

**Figure 1:**
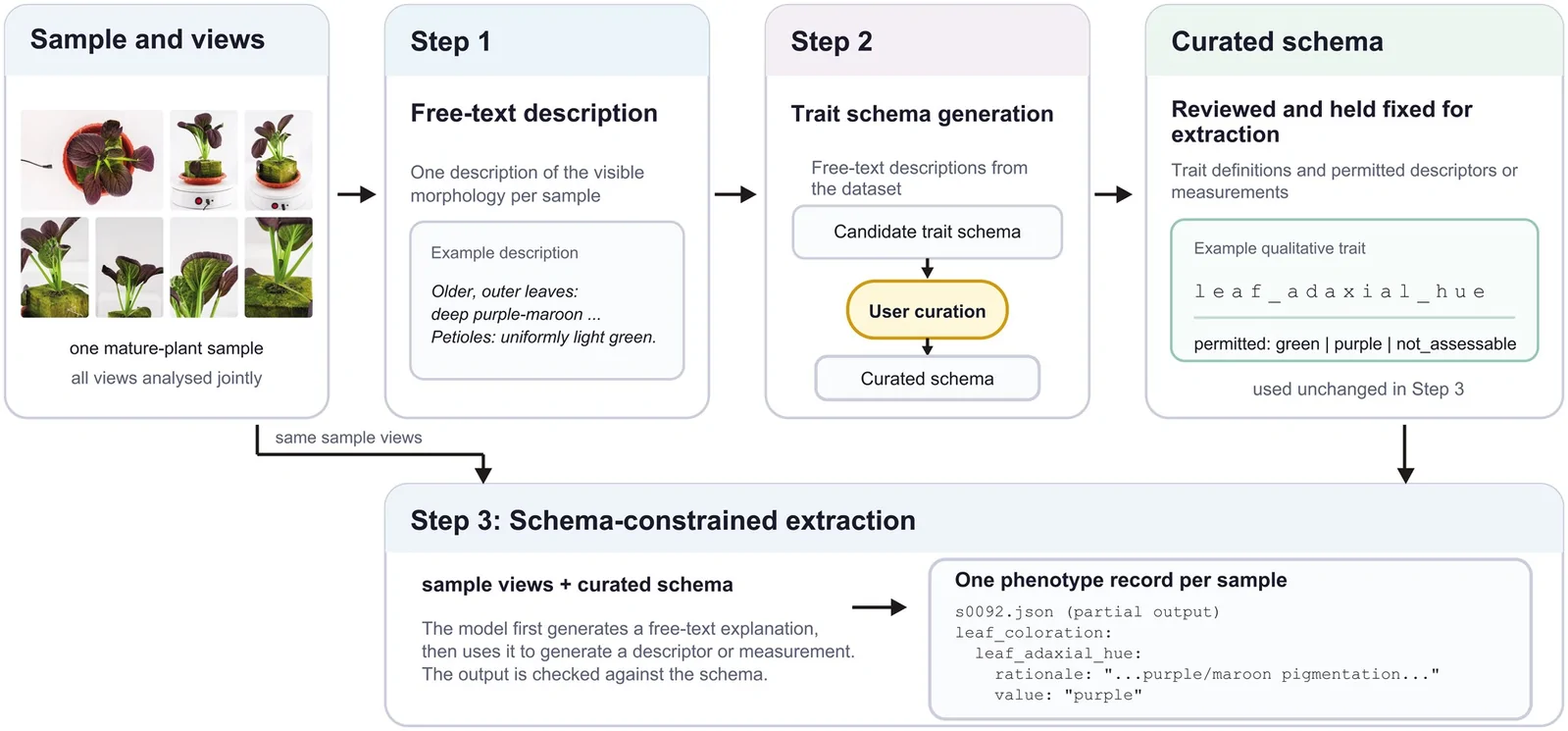
pxGPT workflow, illustrated with one mature plant. All views of a sample are analyzed jointly. In Step 1, a multimodal LLM generates one free-text description of the sample’s visible morphology. In Step 2, descriptions from the image collection are synthesized into a candidate trait schema, which is then reviewed and modified by the user to produce a curated schema. Once finalized, the schema is fixed for subsequent analysis. In Step 3, the same views of each sample are reanalyzed using the fixed schema to generate one structured phenotype record.

In Step 2, descriptions from the image collection are combined to generate a candidate trait schema. Qualitative traits, such as leaf color or margin type, use predefined textual descriptors. Quantitative traits use numerical measurements, with units where appropriate. Each trait includes a definition and guidance for interpreting its permitted descriptors or measurements. The user curates the candidate schema. The curated schema is then fixed and used unchanged for extraction and to generate the human annotation guide. This approach reduces dependence on a predefined trait list but does not eliminate prior assumptions: trait selection remains influenced by model prompts and user decisions. The schema distinguishes biological absence from insufficient visual evidence. The descriptor “absent” means that the relevant structure or feature is not present in a sample that is sufficiently visible for evaluation. The descriptor “not assessable” means that the available views provide insufficient evidence to determine the trait.

In Step 3, each sample is analyzed again using the same views provided in Step 1 and the fixed schema. For each trait, the model first generates a visual-evidence description and then returns a structured output restricted to the permitted descriptors or measurements. pxGPT checks each output against the schema. When a schema contains too many traits for one request, pxGPT processes smaller groups of traits. It verifies that each trait is included exactly once across the groups before combining their outputs into one phenotype record. Extraction performance was evaluated against reference labels derived from independent human annotations. Annotators used the guide generated from the same trait definitions used by pxGPT and had no access to pxGPT predictions or other annotators’ assessments.

The software supports multiple LLM providers, retries failed requests, and reuses completed requests. Current versions record the model provider, model version, schema version, software version, timestamp, and run identifier for each phenotype record. The evaluated runs predated this record-level tracking. For those runs, the model, provider, and timestamp are available at the run level, and the deposited curated schemas document the schema versions.

## 3 Results

We analyzed three *Brassica* datasets with overlapping cultivar representation. The seedling dataset contained samples from 284 cultivars imaged at 2–3 weeks of age. Each sample contained several sibling seedlings of one cultivar and was represented by approximately seven views. The mature_v1 dataset comprised plants from 142 cultivars grown as one synchronized cohort and imaged at 5–6 weeks. The mature_v2 dataset comprised plants from 277 cultivars grown in multiple independent batches over a year and imaged at 5–8 weeks. The labels mature_v1 and mature_v2 indicate collection order rather than distinct developmental stages. In both mature datasets, each sample consisted of one plant represented by ∼20 views spanning side to overhead perspectives. Together, the datasets represented 321 unique cultivars (Table S1, available as supplementary data at *Bioinformatics* online). The curated schemas defined 42, 49, and 50 traits for seedling, mature_v1, and mature_v2, respectively. The primary evaluation included 38, 45, and 43 qualitative traits, respectively. All primary extractions used Claude Sonnet 5.

Ten annotators independently evaluated samples using annotation guides generated from the same curated trait definitions used for pxGPT extraction. They had no access to pxGPT predictions or other annotators’ assessments. In total, they contributed 78 973 annotations across 284 seedling, 112 mature_v1, and 203 mature_v2 samples, a subset of the samples processed by pxGPT. Human reference labels were derived separately for each sample-trait combination. For qualitative traits with unordered descriptors, the most frequent descriptor was used if there was no tie for the highest count. For ordered descriptors, the median was used according to a prespecified rounding rule. This procedure resolved 9946, 4743, and 7828 sample-trait combinations for seedling, mature_v1, and mature_v2, respectively. Unresolved combinations were excluded from performance estimates. Reference labels could be assigned from a single annotation; such combinations included 180 in mature_v1 and 860 in mature_v2. These combinations could not contribute to estimates of agreement among annotators (Table S1, available as supplementary data at *Bioinformatics* online).

Accuracy against human reference labels was calculated separately for each qualitative trait and then averaged across traits. Mean accuracy was 0.619 for seedling, 0.727 for mature_v1, and 0.761 for mature_v2. Table S2, panels B–D, available as supplementary data at *Bioinformatics* online, reports the accuracy estimates and 95% confidence intervals for all 126 dataset-specific qualitative traits. Because traits differed in the number and frequency of their descriptors, we also calculated balanced accuracy, macro-averaged F1, and Cohen’s kappa (Table S2 and Fig. S1, available as supplementary data at *Bioinformatics* online). We also calculated raw pxGPT-human and human-human agreement using the same sample-trait combinations with at least two human annotations, giving each combination equal weight. These combinations were not restricted to resolved reference labels. This weighting differs from the mean accuracy above, which gives each trait equal weight. Agreement between pxGPT and individual annotators was 0.572, 0.691, and 0.712 for seedling, mature_v1, and mature_v2, respectively. Agreement among human annotators was 0.620, 0.739, and 0.730, respectively, and included only combinations with at least two annotations. Differences in raw agreement (pxGPT-human minus human-human) were −0.048, −0.048, and −0.018 for seedling, mature_v1, and mature_v2, respectively. All three 95% confidence intervals were below zero and were estimated from 2000 bootstrap resamples of whole samples (Table S3, available as supplementary data at *Bioinformatics* online). Quantitative traits were evaluated separately using mean absolute error, root mean squared error, and Spearman’s rank correlation (Table S4, available as supplementary data at *Bioinformatics* online).

Across 284 seedling, 142 mature_v1, and 277 mature_v2 samples, pxGPT returned all 32 736 required trait fields (42, 49, and 50 traits per sample, respectively), with 93 empty values and no qualitative descriptor violations (Table S1, available as supplementary data at *Bioinformatics* online). Conformance to the schema does not establish biological correctness. Among resolved sample-trait combinations for traits that permitted absent, pxGPT returned “absent” in 48.8%, 76.3%, and 71.9% of cases in seedling, mature_v1, and mature_v2, respectively. The corresponding frequencies of “not assessable” were 3.7%, 3.2%, and 0.2%.

We then compared these outputs with the human reference labels. Among combinations for which the reference indicated that a structure or feature was present, pxGPT returned “absent” in 8.4%, 25.4%, and 10.9% of cases in seedling, mature_v1, and mature_v2, respectively. Among combinations judged assessable by humans, pxGPT returned “not assessable” in 0.5%, 3.1%, and 0.1% of cases, respectively. Conversely, when the reference label was “not assessable”, pxGPT returned the same descriptor in only 27.1% (88/325), 12.5% (3/24), and 11.1% (5/45) of cases, respectively. Among combinations with an “absent” reference label, false-positive rates were 14.8%, 11.7%, and 13.5%, respectively. When the analysis was restricted to reproductive structures, head or curd structures, and swollen storage organs, the corresponding false-positive rates were 0.6%, 8.0%, and 0.5% (Table S5 and Fig. S2, available as supplementary data at *Bioinformatics* online).

In mature_v2, human reference labels identified 25 plants with at least one reproductive structure, head or curd structure, or swollen storage organ. Some plants were positive for more than one category. Plant-level sensitivity was 1.000 for swollen storage organs (9 positive plants), 0.636 for reproductive structures (11 positive plants), and 0.375 for head or curd structures (8 positive plants). Specificity exceeded 0.98 for each category (Table S6 and Fig. S3, available as supplementary data at *Bioinformatics* online). The small numbers of positive plants limit the precision of the sensitivity estimates; uncertainty is reported using exact binomial confidence intervals. Figure S4, available as supplementary data at *Bioinformatics* online, provides illustrative examples of one corrected classification and one structure that remained undetected.

We tested Step 3 extraction with two additional models using the fixed schemas. GPT 5.6 Luna (OpenAI, USA) processed all 142 mature_v1 samples and included every required trait, with two empty outputs and no qualitative descriptors outside the permitted set. Across the 112 samples with human reference labels, mean accuracy across qualitative traits was 0.736, compared with 0.727 for Claude Sonnet 5. The difference was 0.009 (95% CI, −0.003 to 0.022), estimated by resampling whole plants (Table S7, available as supplementary data at *Bioinformatics* online).

The locally hosted open-weight model Gemma 4 26 b (Google DeepMind, UK) used the same fixed schemas across all three datasets. Differences in mean accuracy relative to Claude Sonnet 5 were 0.021, −0.016, and −0.029 for seedling, mature_v1, and mature_v2, respectively. All three 95% CIs excluded zero (Table S7 and Fig. S5, available as supplementary data at *Bioinformatics* online). These results support reuse of the fixed schemas for Step 3 extraction across the tested models, with accuracy varying by model and dataset. They do not establish equivalent accuracy or reproducibility of the complete pxGPT workflow across models.

Exploratory ablations indicated that both visual-evidence descriptions and trait definitions with their permitted descriptors contributed to extraction accuracy. We detected no accuracy loss when all traits were processed in one request instead of smaller groups (Table S8 and Fig. S6, available as supplementary data at *Bioinformatics* online).

## 4 Conclusion

pxGPT converts multi-view plant images into structured phenotype records using a fixed, curated schema derived from each image collection. Across three *Brassica* datasets, validation compared pxGPT-human agreement with agreement among human annotators. Detection sensitivity varied among morphological structures. Every phenotype record included all required trait fields, although 93 primary-model values were empty; no qualitative descriptor violations were recorded. The same curated schemas were used unchanged across commercial and locally hosted models, and explicit “absent” and “not assessable” descriptors distinguished biological absence from insufficient visual evidence. Within the evaluated growth stages and imaging protocols, these results support pxGPT as a framework for schema-constrained extraction of visible plant morphology.

## Supplementary Material

btag781_Supplementary_Data

## Data Availability

The curated schemas, annotation guides, phenotype records, and human annotations used in this study are available from Zenodo at https://doi.org/10.5281/zenodo.22907554. The source code is available on GitHub at https://github.com/YaoChengLab/pxgpt and an archival version of the source code is available on Zenodo (https://doi.org/10.5281/zenodo.22931745).
