# Supplementary material for: pxGPT: a schema-constrained multimodal large language model framework for plant phenotyping
Source: Bioinformatics. 2026 Oct 20;42(10):btag781. doi: 10.1093/bioinformatics/btag781 (PMC13719227; doi:10.1093/bioinformatics/btag781)
Supplement: Supplementary file 1 [file btag781_supplementary_data.pdf]

# SUPPLEMENTARY NOTES

# pxGPT: A schema-constrained multimodal LLM framework for plant phenotyping

# Supplementary Information

The software is available at https://github.com/YaoChengLab/pxgpt. Curated schemas used in this study, annotation guides, phenotype records and human annotations are available at https://doi.org/10.5281/zenodo.22907554.

# Supplementary Methods

## 1. Datasets, imaging and the scoring unit

We analyzed three overlapping Brassica datasets. The seedling dataset contained 284 cultivars and 1,878 views acquired at 2–3 weeks of age. Each sample comprised a propagation strip containing several sibling seedlings of one cultivar. The strip remained stationary while the photographer moved around it, producing approximately seven views per sample. The mature_v1 dataset contained 142 cultivars and 2,784 views, and the mature_v2 dataset contained 277 cultivars and 5,208 views. The mature_v1 plants were grown as a single synchronized cohort and imaged at 5–6 weeks, whereas the mature_v2 plants were grown in multiple independent batches over a year and imaged at 5–8 weeks. The dataset labels indicate collection order rather than distinct developmental stages. In both mature datasets, each sample comprised one plant photographed on a rotating platform through a full turn, from lateral to directly overhead views, producing approximately 20 views per sample. Across the three datasets, 321 distinct cultivars were represented, with many cultivars occurring in more than one dataset (Supplementary Table S1). Images were captured using a Nikon Z6 camera. A ColorChecker Passport (Calibrite) was included in each session for color calibration. Images were saved in RAW format, color-calibrated using Adobe Lightroom, and exported as JPEG files at a final resolution of 1568 × 1043 pixels.

A sample is the unit analyzed by pxGPT and represented by one phenotype record: a propagation strip of sibling seedlings in the seedling dataset and one plant in each mature dataset. pxGPT receives all available views of a sample jointly rather than analyzing individual views independently. Throughout validation, a sample-trait combination denotes one trait evaluated for one sample. Each dataset therefore contains one sample-trait combination for every eligible pairing of a sample and a trait. This distinction is particularly important for outputs indicating absence, because pxGPT and the annotators evaluated all available views of each sample together.

The curated schemas for the seedling, mature_v1 and mature_v2 datasets contain 42, 49 and 50 traits, respectively. The primary evaluation included the 38, 45 and 43 qualitative traits in these datasets, respectively. The remaining 4, 4 and 7 quantitative traits were analyzed separately because classification performance and measurement error require different evaluation metrics. Human annotations were available for 284 seedling samples, 112 mature_v1 samples and 203 mature_v2 samples (Table S1).

## 2. pxGPT workflow and software implementation

### 2.1 Description, schema synthesis and extraction

pxGPT comprises three steps. In Step 1, a vision-capable multimodal large language model (LLM) receives all views of one sample and generates one free-text description of its visible morphology. Only the final description is retained. In Step 2, a long-context model synthesizes the Step 1 descriptions from the complete image collection into a candidate trait

schema. Recurring qualitative characteristics are represented as qualitative traits with predefined descriptors, whereas quantitative characteristics are represented as quantitative traits with numerical measurements and units where appropriate. Each trait is assigned a scale type, a trait definition and, for qualitative traits, definitions of its permitted descriptors.

The candidate trait schema is then curated by the user before use. The resulting curated schema is assigned a version and fixed before Step 3 extraction and human annotation. This sequence reduces dependence on a predefined trait list but does not eliminate prior assumptions: the Step 1 prompt, model choice, schema-synthesis prompt and user curation can all influence which traits and permitted descriptors or measurements are incorporated into the final schema.

In Step 3, the model receives the same views of each sample together with the fixed schema and performs schema-constrained extraction. It must return a structured output for every required trait, using a permitted descriptor for each qualitative trait or a numerical measurement for each quantitative trait. The primary evaluated runs used Claude Sonnet 5. Step 3 does not receive the Step 1 description. Similar observations in Steps 1 and 3 therefore arise from separate examinations of the same views rather than from transfer of the Step 1 text into the final phenotype record.

### 2.2 Schema compilation and annotation guides

The curated dataset-specific schema serves as the source document and organizes traits by plant organ. Before extraction, the fixed schema is compiled into smaller groups of traits because the complete schema may exceed the grammar complexity supported by constrained decoding or contain JSON Schema constructs that a selected LLM provider cannot enforce.

The compiler divides the curated schema into groups of traits that meet the selected LLM provider's limits for constrained decoding. Traits associated with the same plant organ are kept together unless that group exceeds these limits. For each group, the compiler generates an extraction schema and a human-readable annotation guide from the same curated trait definitions. The extraction schema requires an output field for every trait in the group and prohibits additional fields. It also specifies a short visual-evidence description before the corresponding descriptor or measurement. Qualitative traits use predefined descriptors, whereas quantitative traits represent numerical measurements, with units where applicable. The annotation guide provides the same trait definitions and guidance on descriptors or measurements used for pxGPT extraction.

Compilation stops if a generated extraction schema is invalid, contains a construct unsupported by the selected LLM provider, omits a trait or assigns the same trait to more than one group. The compiler also rejects statements about descriptor frequencies in the annotation instructions because indicating that a particular descriptor is common could bias either pxGPT or human annotators toward that descriptor. The fixed schemas used in the evaluation compiled into 7 groups of traits for the seedling dataset, 10 for mature_v1 and 9 for mature_v2.

The software additionally provides constrained decoding specific to each LLM provider, batch dispatch, caching, checkpointing and retry handling. Current releases record the provider, model identifier, schema name and version, software version, timestamp and run identifier for each phenotype record. The evaluated runs predated record-level provenance tracking. For those runs, provider, model and timestamp were recorded at the run level, while

the deposited curated schemas document the schema versions. These records are sufficient to identify the analyzed artifacts, although they do not permit byte-for-byte reconstruction of every historical model request.

### 2.3 Evidence states

The schema distinguishes absent from not assessable. Absent indicates that the relevant structure or feature is not present in a sample that is otherwise sufficiently visible for evaluation. Not assessable indicates that the available views do not provide sufficient visual evidence to determine the trait, for example because the relevant region is not visible, is occluded or cannot be resolved clearly. Thus, absent represents biological absence, whereas not assessable represents insufficient visual evidence. A missing structured output is a format failure and is not interpreted as either state.

For each trait in Step 3, pxGPT also records a short visual-evidence description of the relevant morphology before assigning the corresponding descriptor or measurement. These descriptions report the visual evidence identified by the model for the corresponding descriptor or measurement and can be inspected when reviewing disagreements or errors. They are not used as an independent source of validation.

## 3. Human annotation and reference construction

Ten annotators were assigned samples from the three image collections. Annotators viewed all available views of each assigned sample and used the annotation guides generated from the fixed schemas. They did not have access to the Step 1 descriptions, Step 3 pxGPT outputs, or other annotators' assessments. The first author did not participate as an annotator. If an annotator submitted more than one set of annotations for the same sample, only the most recent submission was retained.

In total, the annotators provided 78,973 human annotations. Groups of traits were assigned independently, so the number of annotators contributing to a given sample could differ among traits. Each sample-trait combination had between one and four human annotations. All seedling samples were evaluated by two to four annotators, whereas four mature_v1 samples and 20 mature_v2 samples had only one annotator for all assigned traits.

Reference labels were derived separately for each sample-trait combination using only human annotations; pxGPT outputs were not used. For qualitative traits with unordered descriptors, the reference label was the uniquely most frequent descriptor. Combinations in which two or more descriptors were tied for the highest count remained unresolved. Not assessable was included among the permitted descriptors and was assigned as the reference label when it was uniquely most frequent.

For an ordered qualitative (ordinal) trait, not assessable annotations were excluded before constructing the reference label from the ordered descriptors. The permitted descriptors were represented by their ordered levels, and the median level was mapped to the nearest permitted descriptor, with exact halfway cases rounded to the nearest even-numbered level. If all annotations for an ordered qualitative trait were not assessable, not assessable became the reference label. Because the median procedure can produce a descriptor that no individual annotator selected, reference labels for ordered qualitative traits are not described as consensus or majority labels. For a quantitative trait, the reference

measurement was the median of the available human measurements.

A sample-trait combination for an unordered qualitative trait without a unique most frequent descriptor remained unresolved and was excluded from accuracy calculations. The original human annotations and the resolution status of each sample-trait combination were retained in the deposited data. Among qualitative sample-trait combinations eligible for reference construction, 9,946 of 10,792 in the seedling dataset, 4,743 of 5,040 in mature_v1 and 7,828 of 8,729 in mature_v2 had resolved reference labels. The corresponding numbers of unresolved combinations were 846, 297 and 901. Among resolved combinations, 0, 180 and 860, respectively, were based on a single human annotation (Table S1).

## 4. Performance measures

### 4.1 Raw agreement between individual annotators and pxGPT

Raw agreement measures how often two evaluations assign the same descriptor to a sample-trait combination, without correction for chance agreement. We calculated pxGPT-human and human-human agreement using the same eligible sample-trait combinations, each evaluated by at least two human annotators. For each combination, we formed all possible pairs of human annotations and paired the pxGPT output with each individual human annotation.

Because annotation counts varied among sample-trait combinations, pooling all pairs would give greater weight to combinations with more annotations. We therefore calculated agreement within each combination and then averaged these values, giving each combination equal weight.

For sample-trait combination *j*,

$$a_j = \frac{m_j}{q_j}$$

and across *M* eligible sample-trait combinations,

$$A = \frac{1}{M}\sum_{j=1}^{M} a_j.$$

Here, $q_j$ is the number of comparison pairs of the relevant type, $m_j$ is the number of those pairs with matching descriptors, $a_j$ is the corresponding within-combination agreement. *M* is the number of sample-trait combinations eligible for both human-human and pxGPT-human comparisons, and *A* is the reported raw agreement. The calculation was performed separately for pxGPT-human and human-human agreement using the same set of combinations.

Combinations with only one human annotation were excluded from both raw-agreement estimates but could contribute to reference-based accuracy when a reference label was available. As a sensitivity analysis, we also calculated agreement by pooling all comparison pairs directly; this was not the primary estimate.

### 4.2 Accuracy against human reference labels

Reference-based metrics compare the pxGPT descriptor, $\hat{y}_i$, with the human reference label, $y_i$, for each resolved sample-trait combination *i*.

Accuracy is

$$\text{Accuracy} = \frac{1}{N}\sum_{i=1}^{N} I\,(\hat{y}_i = y_i),$$

where $N$ is the number of resolved sample-trait combinations and $I$ equals 1 when the pxGPT descriptor matches the reference label and 0 otherwise. Accuracy is reported separately for each trait with a Wilson 95% confidence interval. The dataset-level summary is the unweighted mean of trait-level accuracies, so each trait contributes equally regardless of the number of resolved sample-trait combinations. The majority-class proportion is reported alongside accuracy as the proportion expected from always assigning the most frequent reference label.

For class-specific metrics, $C$ is defined as the set of classes observed among all resolved human reference labels for a trait, and $K$ is the number of classes in $C$. A descriptor returned by pxGPT that is absent from $C$ is counted as an error for the corresponding reference class, but it is not added as an additional class when averaging performance. Fixing $C$ in this way ensures that the metric definitions remain unchanged across models and bootstrap replicates.

For class $c$, precision and recall are

$$P_c = \frac{TP_c}{TP_c + FP_c}$$

and

$$R_c = \frac{TP_c}{TP_c + FN_c},$$

where $TP_c$ is the number of correctly assigned instances of class $c$, $FP_c$ is the number assigned to $c$ when the reference label is another class, and $FN_c$ is the number with reference label $c$ that were assigned another class. Precision is set to zero when no instances are assigned to class $c$.

The class-specific F1 score is

$$F1_c = \frac{2P_cR_c}{P_c + R_c}$$

The class-specific F1 score is set to zero when both precision and recall are zero and macro-averaged F1 is

$$\text{macro-averaged F1} = \frac{1}{K}\sum_{c\in C} F\,1_c.$$

Macro-averaged F1 was calculated as the unweighted mean of class-specific F1 scores across classes present in the reference labels. Classes with no correct predictions received an F1 score of zero. This metric gives equal weight to each reference class and accounts for both missed instances and false assignments. Its interpretation depends on the reference class frequencies and the distribution of predicted classes.

Balanced accuracy is the mean recall across reference classes:

$$\text{BA} = \frac{1}{K}\sum_{c\in C} R_c.$$

Each reference class therefore contributes equally. A prediction that assigns every sample to a single observed reference class has a balanced accuracy of *1/K*, for example 0.50 for two classes and 0.25 for four classes. Balanced accuracy is therefore interpreted in relation to the number and support of the reference classes.

Weighted F1 is

$$\text{weighted-F1} = \sum_{c \in C} \frac{n_c}{N} F1_c,$$

where $n_c$ is the number of reference labels in class $c$ and $N$ is the total number of evaluated sample-trait combinations. Weighted F1 gives greater weight to more common classes and therefore tends to track overall accuracy more closely than macro-averaged F1.

Cohen's kappa is

$$\kappa = \frac{p_o - p_e}{1 - p_e},$$

where $p_o$ is the observed agreement and $p_e$ is the agreement expected from the marginal class frequencies of the human reference labels and pxGPT outputs. Kappa is calculated over the union of classes used by the human reference and pxGPT because its expected agreement depends on both marginal distributions. Kappa is undefined when $p_e$ = 1.

Four evaluated traits contain only one class in the human reference. Accuracy remains defined for these traits, but balanced accuracy and macro-averaged F1 do not provide information about discrimination among classes. These traits are retained and identified as single-class (Table S2, panels B–D).

For ordered qualitative traits, quadratic-weighted kappa, adjacent-level accuracy and mean absolute level error are also reported. The descriptor not assessable is excluded from these distance-based metrics because it has no position on the ordered descriptor scale.

### 4.3 Quantitative traits

Quantitative traits are evaluated in their original units. For paired pxGPT and human reference measurements, mean absolute error (MAE) and root mean squared error (RMSE) are

$$\text{MAE} = \frac{1}{N} \sum_{i=1}^{N} |y_i - r_i|$$

and

$$\text{RMSE} = \sqrt{\frac{1}{N} \sum_{i=1}^{N} (y_i - r_i)^2}.$$

Here, $y_i$ is the pxGPT measurement, $r_i$ is the human reference measurement and $N$ is the number of paired measurements.

In the seedling dataset, each sample contains several sibling seedlings of one cultivar rather than a single plant. Depending on the trait definition, pxGPT and the annotators therefore recorded either a representative measurement or the maximum measurement across the seedlings visible in the sample. pxGPT measurements and human reference measurements were compared using the same trait-specific definition.

Spearman's rank correlation was used to assess whether pxGPT and the human reference produced similar rankings of samples. Signed mean error was used to assess systematic overestimation or underestimation. Error metrics were calculated separately for

traits with different units; for example, errors expressed in centimeters were not combined with errors expressed as counts.

## 5. Confidence intervals and paired comparisons

All confidence intervals are 95%. Wilson intervals are used for trait-level accuracy because they remain bounded between 0 and 1. Clopper-Pearson exact intervals are used for detection sensitivity, specificity and absence-state rates, for which the underlying counts can be small.

Percentile bootstrap confidence intervals are based on 2,000 replicates. For trait-level balanced accuracy and macro-averaged F1, resolved sample-trait combinations are resampled with replacement while the class set is held fixed to the classes observed in the complete human reference for that trait. If a bootstrap replicate contains no reference instances of one of these classes, that class remains in the calculation, with its recall and F1 set to zero rather than being omitted. This procedure retains sparsely represented classes in the calculation but can produce a conservative lower confidence bound. Confidence intervals for traits with sparse reference classes should be interpreted together with these support measures because resampling cannot add information for classes represented by few samples. The balanced-accuracy bootstrap uses an independent random-number stream with seed `20260912`, ensuring that this calculation does not alter confidence intervals for other metrics. Trait-level kappa and quantitative metrics are likewise bootstrapped by resampling paired sample-trait combinations with replacement.

For the difference between pxGPT-human and human-human raw agreement, whole samples are resampled with replacement. All eligible traits and annotator pairings associated with a selected sample are retained within each bootstrap replicate. Human-human and pxGPT-human raw agreement are then recalculated using the equal-weighting procedure described above, and their difference is recorded. Resampling at the sample level preserves dependence among traits evaluated on the same sample.

Model comparisons are also bootstrapped by resampling whole samples. Within each replicate, trait-level accuracy is recalculated for each model, averaged across the fixed set of traits, and the mean accuracy of the primary model (Claude Sonnet 5) is subtracted from that of the comparison model. McNemar's exact test is reported separately to compare whether each model classified the same sample-trait combinations correctly. The test compares the number of sample-trait combinations classified correctly by only one model with the number classified correctly by only the other. A non-significant result indicates that no difference was detected under this test; it is not evidence that the two models are equivalent.

The component ablation analysis compares paired plant-level accuracies using a paired *t*-test and reports the mean paired difference with a normal-approximation 95% confidence interval. These exploratory comparisons were not adjusted for multiple testing.

## 6. Scoring absent and not assessable states and detecting structures

A trait permitting absent is a qualitative trait whose permitted descriptors include absent. Low levels of a present characteristic are not treated as absent, and developmental-stage traits are excluded because an early developmental stage does not indicate biological absence of the relevant structure. The seedling, mature_v1, and mature_v2 schemas contain 10, 22, and 18 traits permitting absent, respectively.

For each resolved sample-trait combination involving a trait permitting absent, the human reference label and pxGPT output were classified as present, absent, or not assessable. Output frequencies were calculated across all such combinations. A false positive occurred when the human reference label was absent but pxGPT returned a present descriptor; the denominator was all combinations with an absent reference label, including those for which pxGPT returned not assessable. A false absence occurred when the human reference label was present but pxGPT returned absent; the denominator was all combinations with a present reference label. An overly conservative not assessable output occurred when the human reference label was assessable (either present or absent), but pxGPT returned not assessable; the denominator was all combinations with an assessable reference label. A not assessable output matched the reference when both the human reference label and pxGPT output were not assessable. An insufficiently conservative output occurred when the human reference label was not assessable but pxGPT returned either a present descriptor or absent (Table S5).

We also defined a fixed subset of traits representing reproductive structures, head or curd structures, and swollen storage organs. The dataset-specific subsets comprise 2 traits in seedling, 4 in mature_v1, and 3 in mature_v2. False-positive rates at the sample-trait level are calculated using the same definitions as for the complete set of traits permitting absent.

A separate plant-level analysis evaluates whether each structure category was detected at all. A reference-positive plant has at least one category-defining trait for which the human reference label is a descriptor defining presence in that category. pxGPT is considered positive when at least one of the same traits receives a descriptor defining presence in that category. Sensitivity is the proportion of reference-positive plants detected by pxGPT, and specificity is the proportion of reference-negative plants classified as negative. Because a plant can be positive for more than one structure category, counts across categories are not summed (Table S6).

## 7. Portability across LLMs and deployment settings

Comparisons across LLMs and deployment settings used the same curated Step 3 schema, sample views, evaluated traits, and human reference labels for each dataset. Steps 1 and 2 were not repeated because rerunning them could produce different free-text descriptions and, consequently, a different schema.

The curated mature_v1 schema was applied using GPT 5.6 Luna (OpenAI), a second commercial model, for all 142 samples. Schema conformance was evaluated across all 6,958 structured trait outputs. Accuracy was evaluated for the 112 samples with human reference labels, comprising 4,743 resolved sample-trait combinations for qualitative traits. The difference between models in mean accuracy across qualitative traits was evaluated using the sample-level bootstrap described above.

The three curated schemas were also applied using Gemma 4 26b through a locally hosted Ollama service. Schema conformance was evaluated across all samples, and accuracy was evaluated for samples with human reference labels. The released local outputs do not include the exact checkpoint, quantization, runtime, and sampling metadata required for byte-for-byte reproduction. This experiment therefore evaluates whether schema-constrained Step 3 extraction is portable to a locally hosted open-weight LLM; it does not test

portability of the complete three-step workflow or exact reproduction of the historical run.

## 8. Step 3 component ablation

The component study used 119 mature_v2 samples, a subset of the 183 samples with human reference labels supported by at least two annotators. All conditions used GPT 5.6 Luna and the same 50 traits with their permitted descriptors or measurements. Each condition was run twice. Missing structured outputs were counted as incorrect. The full Step 3 configuration was compared with four modified conditions: processing all traits in a single model request rather than in groups of traits; omitting the visual-evidence description; omitting trait and descriptor definitions and the rules for absent and not assessable; and performing extraction from the Step 1 description rather than from the sample views. The text-only condition therefore tests the additional information provided by the views beyond the Step 1 description; it is not a general comparison of text- and vision-based models.

For each plant, accuracy across qualitative traits was averaged over the two runs before comparison with the same plant under the full configuration. Among traits permitting absent, each run contained 1,594 reference-absent and 401 reference-present sample-trait combinations. We measured the false-positive rate for reference-absent combinations, sensitivity for reference-present sample-trait combinations among these traits, and the empty-output rate across 833 quantitative sample-trait combinations per run. Rates were calculated separately for each run and then averaged. Empty outputs were null or empty strings, excluding not assessable. Because all conditions used the same samples and LLM, the study isolates the effects of these Step 3 components within this experimental setting; it does not establish that the observed effect sizes generalize to other models or datasets.

## 9. Conditional trait-dependency sensitivity analysis

Some traits describe characteristics that are meaningful only when another trait indicates that the relevant parent structure is present. For example, flower-petal color is not applicable when no open flower is present. The primary analysis nevertheless scores every required trait because the historical schemas do not encode such dependencies in a machine-readable form.

As a post hoc sensitivity analysis, we reviewed the trait definitions, permitted descriptors or measurements, and design notes in each fixed schema. We identified eight high-confidence and two medium-confidence parent-child trait relationships in which the schema wording explicitly indicated that the child trait should be evaluated only when the parent structure is present. Relationships were not inferred from observed accuracy patterns. For each accepted relationship, the child sample-trait combination was excluded when the human reference label for the parent trait met the specified inapplicability rule. The same reduced set of sample-trait combinations was then used to calculate pxGPT-human and human-human agreement. Table S9 summarizes the effect of these exclusions on mean accuracy against human reference labels.

Because these dependencies were not explicitly encoded in the schemas, the mapping required human interpretation. The analysis is therefore reported as a sensitivity analysis and does not replace the prespecified primary denominator.

# Supplementary Results

## 1. Evaluation scope and annotation support for reference labels

The ten annotators produced 78,973 human annotations across 599 samples with human reference labels. The current analysis uses reference database version v_202609101630. The 38 seedling, 45 mature_v1, and 43 mature_v2 qualitative traits yielded 10,792, 5,040, and 8,729 eligible sample-trait combinations, respectively. Ties among unordered descriptors left 846, 297, and 901 combinations unresolved, leaving 9,946, 4,743, and 7,828 resolved sample-trait combinations for reference-based metrics (Table S1).

The human reference labels have two important limitations. First, 180 mature_v1 and 860 mature_v2 resolved sample-trait combinations are supported by only one annotator and therefore provide no human-human comparison for that combination. Excluding all single-annotator combinations does not change mean accuracy across qualitative traits in seedling, which has no such combinations; it changes mean accuracy by less than 0.001 in mature_v1 and from 0.761 to 0.767 in mature_v2. Second, the median rule for ordered qualitative traits determines the human reference label for 1,686, 251, and 585 sample-trait combinations in seedling, mature_v1, and mature_v2, respectively, and can assign a descriptor that was not selected by any individual annotator. Excluding these combinations changes mean accuracy across qualitative traits to 0.636, 0.736, and 0.771, respectively. Alternative half-up and half-down rounding rules change seedling accuracy over a range of 0.023. These sensitivity analyses are reported to distinguish the effects of the reference-label aggregation rules from direct biological adjudication. Table S9 summarizes the rounded-median exclusions and alternative rounding rules. Across the alternative reference-label rules, balanced accuracy changed by at most 0.0102 and macro-F1 by at most 0.0170 relative to the primary rule (Table S9, panel A).

## 2. Performance for qualitative traits and human-human agreement

Using the same eligible sample-trait combinations with at least two human annotations and giving each combination equal weight, raw pxGPT-human agreement was 0.572 for seedling, 0.691 for mature_v1, and 0.712 for mature_v2. Raw human-human agreement was 0.620, 0.739, and 0.730, respectively. The corresponding differences, calculated as pxGPT-human minus human-human agreement, were −0.048, −0.048, and −0.018, with sample-resampled 95% confidence intervals of −0.060 to −0.035, −0.060 to −0.036, and −0.027 to −0.010 (Table S3). These measures quantify exact agreement between pxGPT and individual annotators or between pairs of annotators. Human-human agreement provides context for the reproducibility of human annotation but is not a performance ceiling. These comparisons do not establish equivalence.

Mean accuracy across qualitative traits against the human reference labels was 0.619, 0.727, and 0.761 for seedling, mature_v1, and mature_v2, respectively. The corresponding mean majority-class proportions were 0.610, 0.745, and 0.766, illustrating why accuracy alone can be difficult to interpret when descriptor frequencies are imbalanced. Across the 126 dataset-specific qualitative traits, the human reference labels contained between one and seven observed classes: 29 traits had two classes, 41 had three, 40 had four, 12 had at least five, and four had only one (Fig. S1). Table S2, panels B–D, reports per-trait accuracy with 95% confidence intervals and the number of resolved sample-trait combinations. Table S2, panels E–G, summarizes the distributions of balanced accuracy, macro-averaged F1 and

Cohen’s kappa across traits, together with reference-class counts, class balance and support.

All 32,736 required trait fields were present across the three primary phenotype-record sets, with 93 empty values and no qualitative descriptor violations (Table S1). This demonstrates conformance to the schema-constrained output format; it does not imply that every permitted descriptor returned by pxGPT was biologically correct.

## 3. Quantitative trait performance

The four seedling, four mature_v1, and seven mature_v2 quantitative traits were analyzed separately (Table S4). Performance varied among traits. For each trait, the table reports mean absolute error, root mean squared error, signed mean error, and Spearman’s rank correlation. Because measurements expressed as counts and centimeters are not directly comparable, error metrics were not aggregated across traits.

## 4. Absent and not assessable states and structure detection

Across the 10, 22, and 18 traits permitting absent, pxGPT returned absent for 48.8%, 76.3%, and 71.9% of resolved sample-trait combinations in seedling, mature_v1, and mature_v2, respectively. It returned not assessable for 3.7%, 3.2%, and 0.2%. Among combinations for which the human reference indicated presence, false-absence rates were 8.4%, 25.4%, and 10.9%. Across all combinations with an assessable human reference label, rates of overly conservative not assessable outputs were 0.5%, 3.1%, and 0.1%. Conversely, among combinations whose human reference label was itself not assessable, pxGPT also returned not assessable in 27.1% (88/325), 12.5% (3/24), and 11.1% (5/45) of cases and otherwise returned a descriptor indicating presence or absence. Among combinations with an absent human reference label, false-positive rates were 14.8%, 11.7%, and 13.5%. Supplementary Table S5 reports these outcomes with their counts, denominators, and intervals.

False-positive rates were lower for the fixed subset of traits representing reproductive structures, head or curd structures, and swollen storage organs: 0.6% in seedling, 8.0% in mature_v1, and 0.5% in mature_v2. The mature_v1 dataset contained only four reference-positive plant-category combinations across three plants, so its structure-detection results are informative primarily about specificity. In contrast, mature_v2 contained 25 distinct plants with a human reference label indicating the presence of at least one of these structure categories.

In mature_v2, pxGPT detected swollen storage organs in all 9 reference-positive plants, reproductive structures in 7 of 11 reference-positive plants, and head or curd structures in 3 of 8 reference-positive plants. The corresponding sensitivities were 1.000, 0.636, and 0.375, with exact 95% confidence intervals of 0.664 to 1.000, 0.308 to 0.891, and 0.085 to 0.755. Specificity exceeded 0.98 for each category (Table S6; Figure S3). Restricting reference-positive cases to those supported by at least two annotators retained complete detection of swollen storage organs but did not eliminate the lower sensitivity for head or curd structures.

Table S6, panel B, summarizes nine category-detection failures and one storage-organ identity error, with condensed model-reported visual evidence for each record. Three source descriptions end with ellipses; the complete original responses are not supplied. These descriptions report the visual evidence identified by the model and should not be interpreted as explanations of why an error occurred. Among the recorded cases, four descriptions

applied a stricter visual criterion than the human reference label, four stated that the relevant feature was not visible, and two identified a different structure. These categories summarize the model-generated text and are not interpreted as causal failure modes.

Figure S4 presents two illustrative cases rather than an additional performance estimate. For cultivar s0266, the Step 1 description noted a pale, thickened hypocotyl but did not identify a swollen storage organ, whereas Step 3 returned turnip_swollen_hypocotyl, matching both annotators. For cultivar s0170, both Step 1 and Step 3 missed a reproductive stem and terminal buds recorded by four annotators. These examples show that the classification from schema-constrained extraction can differ from that in the preceding free-text description, but that a structure can remain undetected in both steps. Step 3 independently re-examines the images; it does not use or revise the Step 1 text.

## 5. Portability across LLMs and deployment settings

The second commercial LLM, GPT 5.6 Luna, returned all 49 required trait fields for all 142 mature_v1 samples, with two empty values and no qualitative descriptor violations. The primary model (Claude Sonnet 5) returned all required fields with no empty values or descriptor violations (Table S7). Among the 112 samples with human reference labels, mean accuracy across qualitative traits was 0.736 for the second model and 0.727 for the primary model. The difference was 0.9 percentage points, with a sample-resampled 95% confidence interval of −0.3 to 2.2 percentage points. McNemar's exact test of paired correctness across the 4,743 resolved sample-trait combinations gave $p = 0.23$. Because the confidence interval included zero, this comparison did not detect a difference in mean accuracy across qualitative traits between the two models; it does not establish equivalence.

The locally hosted open-weight LLM, Gemma 4 26b, returned every required trait field with no qualitative descriptor violations; 68, 12, and 106 values were empty in seedling, mature_v1, and mature_v2, respectively. Mean accuracy across qualitative traits was 0.640 for seedling, 0.711 for mature_v1, and 0.732 for mature_v2, compared with 0.619, 0.727, and 0.761 for the primary model, respectively. The differences between the local and primary models (local minus primary) were 2.1 percentage points (95% confidence interval, 0.9 to 3.2), −1.6 (−2.8 to −0.4), and −2.9 (−3.8 to −2.0), respectively (Table S7; Figure S5). The direction of the difference therefore varied among datasets. These results support portability of schema-constrained Step 3 extraction across the tested LLMs, but do not establish portability of the complete three-step workflow or exact reproduction of the historical runs.

## 6. Contribution of individual components

Across two replicate runs, the full configuration achieved a mean plant-level accuracy of 0.707 for qualitative traits. Removing the visual-evidence description reduced accuracy to 0.680, a difference of −2.68 percentage points (95% confidence interval, −3.57 to −1.79), and increased the false-positive rate for absent structures from 0.266 to 0.321. Removing trait and descriptor definitions and the rules for absent and not assessable reduced accuracy to 0.692, a difference of −1.42 percentage points (95% confidence interval, −2.52 to −0.32). These findings support the visual-evidence description and the combined definitions and scoring rules as components of the evaluated implementation (Table S8; Figure S6).

Processing all traits in a single request yielded an accuracy of 0.715, a difference of 0.84 percentage points relative to the full configuration (95% confidence interval, –0.06 to 1.75). Thus, the study did not detect an accuracy loss when grouping was removed; however, processing traits in groups may still be needed to accommodate model grammar constraints and control request size. The text-only condition achieved an accuracy across qualitative traits of 0.718, but an average of 83.1% of its quantitative sample-trait combinations had empty outputs, compared with 4.0% under the full configuration. Mean sensitivity across 401 sample-trait combinations per run with reference labels indicating structure presence decreased from 0.976 to 0.796. Accuracy across qualitative traits alone therefore did not capture the information lost when views were omitted. These exploratory analyses were limited to 119 plants from mature_v2 and one LLM (Table S8).

## 7. Sensitivity analyses and limitations

The post hoc analysis of conditional traits identified ten dependencies between parent and child traits from the written definitions in the fixed schemas. Excluding a child trait when the human reference label for its parent met the specified inapplicability rule removed 6.06%, 4.62%, and 5.17% of qualitative sample-trait combinations from the seedling, mature_v1, and mature_v2 datasets, respectively. Mean accuracy across qualitative traits changed from 0.619 to 0.610 for seedling, from 0.727 to 0.728 for mature_v1, and from 0.761 to 0.746 for mature_v2. The effect was concentrated in a small number of traits. For example, accuracy for leaf anthocyanin distribution in mature_v2 decreased from 0.667 to 0.302. Among traits retaining at least 20 sample-trait combinations, the difference between pxGPT-human and human-human agreement changed by no more than 0.3 percentage points. Because these dependencies were inferred retrospectively from written definitions and were not machine-readable, these estimates are presented as a sensitivity analysis rather than as the primary results (Table S9).

The validation has four additional limitations. First, sample-trait combinations with unresolved ties among unordered descriptors lacked resolved human reference labels and were excluded from all accuracy estimates. Second, 3.8% of resolved sample-trait combinations in mature_v1 and 11.0% in mature_v2 relied on a single annotator. Third, rare reference classes yielded imprecise class-specific metrics, although the interval method preserved the complete class set. Fourth, comparisons among models used the same fixed schema for Step 3 and therefore did not evaluate schema generation by other models. More broadly, all estimates apply to visible *Brassica* morphology at the sampled developmental stages and under the specified imaging protocols. Transfer to other taxa, developmental stages, or image-acquisition protocols was not evaluated.

# Supplementary Tables

## Supplementary Table S1. Datasets and human reference labels.

| Measure | seedling | mature_v1 | mature_v2 |
|---|---|---|---|
| Sample unit | Sibling-seedling strip | One plant | One plant |
| Age at imaging (weeks) | 2-3 | 5-6 | 5-8 |
| Cultivars | 284 | 142 | 277 |
| Samples processed | 284 | 142 | 277 |
| Images | 1,878 | 2,784 | 5,208 |
| Human-annotated samples | 284 | 112 | 203 |
| Qualitative traits | 38 | 45 | 43 |
| Quantitative traits | 4 | 4 | 7 |
| Eligible qualitative combinations | 10,792 | 5,040 | 8,729 |
| Resolved reference combinations | 9,946 | 4,743 | 7,828 |
| Unresolved ties (unordered traits) | 846 | 297 | 901 |
| Resolved combinations with one annotator | 0 | 180 | 860 |
| Required primary-model fields | 11,928 | 6,958 | 13,850 |
| Missing primary-model fields | 0 | 0 | 0 |
| Empty primary-model values | 91 | 0 | 2 |
| Qualitative descriptor violations | 0 | 0 | 0 |

- A seedling sample contains sibling seedlings of one cultivar; a mature sample is one plant. All views of each sample are assessed jointly. mature_v1 was one synchronized cohort; mature_v2 comprised independent batches over a year. The labels indicate collection order.
- Cultivars overlap among datasets: 321 unique cultivars in total. Ten annotators contributed 78,973 annotations across 599 samples. Qualitative reference labels use the unique mode for unordered traits and the median with half-to-even rounding for ordered traits.
- Primary model: Claude Sonnet 5. All 32,736 required fields were present; 93 values were empty. A missing field, an empty value, and a not assessable descriptor are distinct outcomes.

## Supplementary Table S2. Qualitative-trait accuracy, score distributions and reference-class support.

### A. Dataset summary

| Dataset | Traits | Resolved pairs | Mean accuracy | Mean majority-class share | Mean balanced accuracy | Mean macro-F1 |
|---|---|---|---|---|---|---|
| seedling | 38 | 9,946 | 0.619 | 0.610 | 0.547 | 0.501 |
| mature_v1 | 45 | 4,743 | 0.727 | 0.745 | 0.611 | 0.536 |
| mature_v2 | 43 | 7,828 | 0.761 | 0.766 | 0.592 | 0.555 |

- Metrics compare pxGPT with resolved human reference labels. Dataset summaries give each trait equal weight. Majority-class share is the fraction of labels in the largest reference class, averaged across traits.
- Results summarize 126 dataset-specific qualitative traits. Single-class trait counts are 0, 1, and 3 in dataset order. No dataset interval is obtained by averaging trait intervals. Panels B–D report per-trait accuracy and 95% confidence intervals, with flags for limited class support.
- n = resolved sample-trait combinations. Accuracy uses a 0–1 scale and is followed by its Wilson 95% confidence interval. Values are rounded to three decimal places.
- Table S2, panels E–G, summarizes the distributions of balanced accuracy, macro-averaged F1 and Cohen's kappa across traits, together with reference-class counts, class balance and support.
- A reference class has only one pair; uncertainty for that class cannot be estimated reliably. † The reference has one class. These flags follow the source results.

## Supplementary Table S2 (continued). B. seedling: 38 qualitative traits.

n = resolved combinations. * One-pair reference class; † single-class reference.

| Trait | n | Accuracy [95% CI] |
|---|---|---|
| **Cotyledon** | | |
| cotyledon anthocyanin presence | 225 | 0.236 [0.185–0.295] |
| cotyledon persistence senescence | 284 | 0.310 [0.259–0.366] |
| cotyledon shape | 214 | 0.196 [0.149–0.255] |
| **Hypocotyl stem** | | |
| hypocotyl stem anthocyanin intensity | 284 | 0.662 [0.605–0.715] |
| hypocotyl stem elongation | 284 | 0.500 [0.442–0.558] |
| stem base swelling* | 258 | 0.957 [0.925–0.976] |
| **Leaf apex base** | | |
| leaf apex shape | 215 | 0.805 [0.747–0.852] |
| leaf base shape | 217 | 0.747 [0.685–0.800] |
| **Leaf blade** | | |
| leaf blade anthocyanin coverage | 284 | 0.592 [0.534–0.647] |
| leaf blade anthocyanin intensity | 284 | 0.440 [0.384–0.498] |
| leaf blade anthocyanin surface | 223 | 0.596 [0.531–0.659] |
| leaf blade green intensity | 284 | 0.609 [0.551–0.664] |
| leaf blade lobing | 284 | 0.655 [0.598–0.708] |
| leaf blade shape* | 195 | 0.426 [0.358–0.496] |
| **Leaf margin** | | |
| leaf margin type | 185 | 0.449 [0.379–0.521] |
| **Leaf surface** | | |
| leaf surface glaucousness | 284 | 0.616 [0.558–0.671] |
| leaf surface glossiness | 210 | 0.700 [0.635–0.758] |
| leaf surface rugosity | 284 | 0.606 [0.548–0.661] |
| leaf trichome density | 284 | 0.845 [0.798–0.883] |
| leaf trichome distribution | 284 | 0.768 [0.715–0.813] |

| Trait | n | Accuracy [95% CI] |
|---|---|---|
| **Petiole** | | |
| petiole anthocyanin intensity | 284 | 0.630 [0.573–0.684] |
| petiole form | 240 | 0.688 [0.626–0.743] |
| petiole relative length | 284 | 0.521 [0.463–0.579] |
| **Phenology** | | |
| developmental stage | 284 | 0.493 [0.435–0.551] |
| flower color hue* | 282 | 0.989 [0.969–0.996] |
| reproductive status* | 271 | 0.937 [0.902–0.961] |
| **Plant health** | | |
| foliar lesion presence* | 224 | 0.652 [0.587–0.711] |
| leaf turgor | 284 | 0.775 [0.723–0.819] |
| plant senescence degree | 284 | 0.405 [0.350–0.463] |
| **Root system** | | |
| root color hue | 237 | 0.738 [0.679–0.790] |
| root density | 284 | 0.486 [0.428–0.544] |
| root hair density | 284 | 0.486 [0.428–0.544] |
| **Venation** | | |
| midrib anthocyanin intensity | 284 | 0.655 [0.598–0.708] |
| midrib prominence | 284 | 0.461 [0.404–0.519] |
| secondary vein pattern | 240 | 0.883 [0.837–0.918] |
| secondary vein prominence | 284 | 0.722 [0.667–0.771] |
| **Whole plant architecture** | | |
| leaf posture | 284 | 0.602 [0.544–0.657] |
| plant growth habit | 262 | 0.687 [0.629–0.740] |

## Supplementary Table S2 (continued). C. mature_v1: 45 qualitative traits.

n = resolved combinations. * One-pair reference class; † single-class reference.

| Trait | n | Accuracy [95% CI] |
|---|---|---|
| **Foliar condition** | | |
| leaf interveinal chlorosis† | 108 | 0.963 [0.909–0.986] |
| leaf necrotic lesions | 109 | 0.908 [0.839–0.949] |
| leaf variegation | 110 | 0.900 [0.830–0.943] |
| **Inflorescence** | | |
| flower petal color hue | 111 | 0.946 [0.887–0.975] |
| fruit silique presence* | 111 | 0.991 [0.951–0.998] |
| inflorescence curd formation* | 108 | 0.991 [0.949–0.998] |
| inflorescence stage* | 112 | 0.902 [0.833–0.944] |
| **Leaf blade** | | |
| leaf blade anthocyanin coverage | 112 | 0.705 [0.615–0.782] |
| leaf blade apex shape | 99 | 0.849 [0.765–0.906] |
| leaf blade base shape | 87 | 0.609 [0.504–0.705] |
| leaf blade curvature | 94 | 0.404 [0.311–0.505] |
| leaf blade green intensity | 112 | 0.607 [0.515–0.693] |
| leaf blade shape* | 87 | 0.563 [0.459–0.663] |
| leaf heterophylly presence | 112 | 0.920 [0.854–0.957] |
| **Leaf damage** | | |
| leaf damage extent | 112 | 0.679 [0.587–0.758] |
| leaf damage type | 110 | 0.682 [0.590–0.762] |
| **Leaf margin** | | |
| leaf margin anthocyanin | 105 | 0.867 [0.789–0.919] |
| leaf margin type | 87 | 0.598 [0.493–0.695] |
| **Leaf surface** | | |
| leaf abaxial anthocyanin | 108 | 0.296 [0.218–0.388] |
| leaf surface glaucousness | 112 | 0.661 [0.569–0.742] |
| leaf surface pubescence | 110 | 0.918 [0.852–0.956] |

| Trait | n | Accuracy [95% CI] |
|---|---|---|
| **Petiole** | | |
| petiole anthocyanin | 105 | 0.810 [0.724–0.873] |
| petiole cross section shape | 102 | 0.824 [0.738–0.885] |
| petiole relative length | 112 | 0.518 [0.426–0.608] |
| petiole thickness | 112 | 0.598 [0.506–0.684] |
| **Phenology** | | |
| cotyledon persistence* | 102 | 0.951 [0.890–0.979] |
| foliar senescence* | 112 | 0.768 [0.682–0.836] |
| plant developmental stage* | 112 | 0.929 [0.865–0.963] |
| **Root system** | | |
| root colonization extent | 112 | 0.536 [0.444–0.625] |
| root color | 104 | 0.779 [0.690–0.848] |
| root density | 112 | 0.634 [0.542–0.717] |
| root hair visibility | 103 | 0.583 [0.486–0.673] |
| **Stem** | | |
| stem base anthocyanin | 98 | 0.806 [0.717–0.872] |
| stem elongation | 112 | 0.705 [0.615–0.782] |
| stem leaf scars | 89 | 0.360 [0.268–0.463] |
| stem surface texture | 99 | 0.707 [0.611–0.788] |
| stem thickness | 112 | 0.384 [0.299–0.476] |
| **Venation** | | |
| leaf vein anthocyanin | 107 | 0.916 [0.848–0.955] |
| leaf venation pattern | 97 | 0.804 [0.714–0.871] |
| **Whole plant architecture** | | |
| leaf phyllotaxy* | 86 | 0.570 [0.464–0.669] |
| plant axillary bud development* | 109 | 0.927 [0.862–0.962] |
| plant branching habit | 111 | 0.964 [0.911–0.986] |

| Trait | n | Accuracy [95% CI] |
|---|---|---|
| leaf surface texture* | 112 | 0.554 [0.461–0.642] |

| Trait | n | Accuracy [95% CI] |
|---|---|---|
| plant growth habit | 96 | 0.333 [0.247–0.432] |
| plant head formation* | 111 | 0.784 [0.698–0.850] |

## Supplementary Table S2 (continued). D. mature_v2: 43 qualitative traits.

n = resolved combinations. * One-pair reference class; † single-class reference.

| Trait | n | Accuracy [95% CI] |
|---|---|---|
| **Leaf apex base** | | |
| leaf apex shape | 190 | 0.942 [0.899–0.967] |
| leaf base shape | 130 | 0.423 [0.342–0.509] |
| **Leaf blade** | | |
| heterophylly | 195 | 0.785 [0.722–0.837] |
| leaf blade shape | 110 | 0.646 [0.553–0.729] |
| **Leaf coloration** | | |
| leaf adaxial hue | 200 | 0.985 [0.957–0.995] |
| leaf anthocyanin distribution* | 156 | 0.667 [0.590–0.736] |
| leaf anthocyanin extent | 203 | 0.759 [0.695–0.812] |
| leaf anthocyanin hue | 173 | 0.827 [0.763–0.876] |
| leaf green intensity | 203 | 0.724 [0.659–0.781] |
| leaf variegation | 169 | 0.852 [0.791–0.898] |
| **Leaf margin** | | |
| leaf margin dentition | 137 | 0.745 [0.666–0.810] |
| leaf margin undulation | 203 | 0.286 [0.228–0.351] |
| **Leaf surface** | | |
| leaf bullation | 203 | 0.769 [0.706–0.821] |
| leaf glaucousness | 203 | 0.562 [0.493–0.628] |
| leaf pubescence | 203 | 0.951 [0.912–0.973] |
| **Petiole** | | |
| petiole color hue | 168 | 0.869 [0.810–0.912] |
| petiole cross section | 159 | 0.610 [0.533–0.682] |
| petiole form | 182 | 0.857 [0.799–0.901] |
| **Phenology** | | |
| bolting status | 203 | 0.975 [0.944–0.989] |
| developmental stage | 203 | 0.764 [0.701–0.817] |
| **Physiological status** | | |
| interveinal chlorosis | 198 | 0.859 [0.803–0.900] |
| leaf disc sampling marks† | 203 | 0.990 [0.965–0.997] |
| marginal tipburn necrosis | 182 | 0.780 [0.715–0.834] |
| mechanical damage† | 197 | 0.965 [0.929–0.983] |
| pest or disease signs* | 197 | 0.777 [0.714–0.829] |
| senescence extent* | 203 | 0.680 [0.613–0.740] |
| wilting turgor loss* | 202 | 0.985 [0.957–0.995] |
| **Root system** | | |
| root color hue | 151 | 0.894 [0.835–0.934] |
| root extrusion | 203 | 0.571 [0.503–0.638] |
| root visibility* | 179 | 0.916 [0.866–0.949] |
| **Stem** | | |
| internode elongation | 203 | 0.704 [0.638–0.763] |
| stem anthocyanin extent | 203 | 0.645 [0.577–0.708] |
| **Substrate** | | |
| growing medium† | 202 | 1.000 [0.981–1.000] |
| substrate algae | 115 | 0.783 [0.699–0.848] |
| substrate fungal growth | 182 | 0.643 [0.571–0.709] |
| **Venation** | | |
| midrib prominence | 203 | 0.522 [0.454–0.590] |
| vein color hue* | 152 | 0.632 [0.553–0.704] |
| vein lamina contrast | 203 | 0.749 [0.685–0.803] |
| **Whole plant architecture** | | |
| leaf posture | 159 | 0.239 [0.179–0.311] |
| plant growth habit | 119 | 0.454 [0.367–0.543] |
| storage organ | 195 | 0.990 [0.963–0.997] |

| Trait | n | Accuracy [95% CI] |
|---|---|---|
| reproductive or head structure | 189 | 0.963 [0.926–0.982] |

| Trait | n | Accuracy [95% CI] |
|---|---|---|
| storage organ surface color* | 195 | 0.980 [0.949–0.992] |

## Supplementary Table S2 (continued). Reference-class support and score distributions.

### E. Number of traits by observed reference-class count

| Dataset | Traits | 1 class | 2 classes | 3 classes | 4 classes | ≥5 classes | Any class n = 1 |
|---|---|---|---|---|---|---|---|
| seedling | 38 | 0 | 1 | 8 | 25 | 4 | 5 |
| mature_v1 | 45 | 1 | 19 | 15 | 7 | 3 | 11 |
| mature_v2 | 43 | 3 | 9 | 18 | 8 | 5 | 7 |

### F. Class balance and smallest reference-class support across traits

| Dataset | Majority-class share Median [Q1, Q3] | Share range | Smallest class (combinations) Median [Q1, Q3] | Support range |
|---|---|---|---|---|
| seedling | 0.586 [0.464, 0.715] | 0.351–0.997 | 10.5 [2.5, 16.8] | 1.0–49.0 |
| mature_v1 | 0.760 [0.570, 0.964] | 0.368–1.000 | 3.0 [2.0, 11.0] | 1.0–108.0 |
| mature_v2 | 0.788 [0.604, 0.948] | 0.399–1.000 | 5.0 [2.5, 12.5] | 1.0–203.0 |

### G. Score distributions across traits

| Dataset | Metric | Traits with defined scores | Median [Q1, Q3] | Range |
|---|---|---|---|---|
| seedling | Balanced accuracy | 38 | 0.560 [0.446, 0.614] | 0.275–0.995 |
| seedling | Macro-F1 | 38 | 0.526 [0.419, 0.585] | 0.150–0.831 |
| seedling | Cohen's kappa | 38 | 0.317 [0.225, 0.418] | 0.035–0.713 |
| mature_v1 | Balanced accuracy | 45 | 0.620 [0.473, 0.736] | 0.250–0.967 |
| mature_v1 | Macro-F1 | 45 | 0.511 [0.456, 0.618] | 0.182–0.981 |
| mature_v1 | Cohen's kappa | 45 | 0.233 [0.082, 0.439] | -0.037–0.678 |
| mature_v2 | Balanced accuracy | 43 | 0.563 [0.457, 0.715] | 0.201–1.000 |
| mature_v2 | Macro-F1 | 43 | 0.507 [0.420, 0.684] | 0.134–1.000 |
| mature_v2 | Cohen's kappa | 42 | 0.320 [0.068, 0.578] | -0.028–0.891 |

- Each trait contributes one value within its dataset. Panel E counts classes observed in the human reference labels; the last column counts traits with at least one class represented by only one resolved sample-trait pair. Panel F summarizes the majority-class proportion and the smallest reference-class count for each trait.
- Q1 and Q3 are the 25th and 75th percentiles, calculated by linear interpolation. These quartiles describe variation across traits, not confidence intervals. Panel G includes single-class traits where a score is defined; these traits do not test discrimination among reference classes. Cohen's kappa is undefined for one mature_v2 trait (growing_medium), leaving 42 defined values. Per-trait accuracy and 95% confidence intervals are in Table S2.

## Supplementary Table S3. Agreement with individual annotators.

| Dataset | Samples | Eligible pairs | Human-human agreement | pxGPT-human agreement | Difference [95% CI] |
|---|---|---|---|---|---|
| seedling | 284 | 10,792 | 0.620 | 0.572 | -0.048 [-0.060, -0.035] |
| mature_v1 | 108 | 4,860 | 0.739 | 0.691 | -0.048 [-0.060, -0.036] |
| mature_v2 | 183 | 7,869 | 0.730 | 0.712 | -0.018 [-0.027, -0.010] |

- Both estimates use the same sample-trait combinations with at least two human annotations. Each combination has equal weight. These combinations are not restricted to resolved reference labels; their denominators therefore differ from those in Table S2.
- Difference = pxGPT-human minus human-human agreement. Intervals use 2,000 bootstrap resamples of whole samples. Human-human agreement provides context, not a performance ceiling.

## Supplementary Table S4. Errors for quantitative traits.

| Dataset | Trait (unit) | Numeric pairs / reference pairs | MAE [95% CI] | RMSE | Signed mean error | Spearman rho [95% CI] |
|---|---|---|---|---|---|---|
| seedling | leaf blade length (cm) | 266/284 | 1.282 [1.157, 1.412] | 1.686 | 0.831 | 0.465 [0.357, 0.561] |
| seedling | plant height (cm) | 274/284 | 1.350 [1.212, 1.500] | 1.832 | -0.364 | 0.600 [0.509, 0.682] |
| seedling | root length (cm) | 207/284 | 1.141 [0.987, 1.320] | 1.657 | -0.865 | 0.475 [0.349, 0.586] |
| seedling | true leaf count (count) | 271/284 | 1.437 [1.260, 1.638] | 2.124 | -0.445 | 0.471 [0.367, 0.568] |
| mature_v1 | leaf blade length (cm) | 112/112 | 2.378 [2.032, 2.744] | 3.046 | 1.964 | 0.614 [0.483, 0.720] |
| mature_v1 | plant canopy spread (cm) | 112/112 | 4.124 [3.518, 4.749] | 5.274 | 2.742 | 0.696 [0.578, 0.785] |
| mature_v1 | plant height (cm) | 112/112 | 2.858 [2.381, 3.369] | 3.935 | 1.261 | 0.785 [0.682, 0.857] |
| mature_v1 | plant true leaf number (count) | 112/112 | 1.772 [1.295, 2.371] | 3.301 | -1.549 | 0.854 [0.784, 0.902] |
| mature_v2 | canopy spread (cm) | 203/203 | 4.347 [3.797, 4.984] | 6.164 | -0.827 | 0.715 [0.626, 0.786] |
| mature_v2 | leaf count (count) | 203/203 | 1.739 [1.369, 2.200] | 3.562 | -1.261 | 0.785 [0.721, 0.838] |
| mature_v2 | leaf length (cm) | 203/203 | 4.303 [3.904, 4.730] | 5.255 | 3.953 | 0.661 [0.568, 0.735] |
| mature_v2 | leaf width (cm) | 203/203 | 1.425 [1.275, 1.580] | 1.821 | 0.603 | 0.695 [0.611, 0.766] |
| mature_v2 | petiole length (cm) | 200/203 | 1.864 [1.596, 2.155] | 2.740 | 0.393 | 0.737 [0.657, 0.800] |
| mature_v2 | plant height (cm) | 203/203 | 2.749 [2.446, 3.089] | 3.677 | -0.610 | 0.792 [0.732, 0.841] |
| mature_v2 | stem diameter (cm) | 196/203 | 0.726 [0.538, 0.983] | 1.703 | -0.432 | 0.521 [0.395, 0.627] |

- MAE = mean absolute error; RMSE = root mean squared error. Both errors and signed mean error use the trait unit. Signed error = pxGPT minus the median human reference. Correlation measures ranking, not absolute measurement error.
- All 15 quantitative traits are retained. Errors are not pooled across traits or units. Intervals use paired sample-trait bootstrap resampling. The seedling unit is a strip of sibling seedlings; the trait definition specifies a representative or maximum measurement. Reasons for every unpaired value are not supplied.

## Supplementary Table S5. Absent and not assessable outcomes.

| Outcome | Human reference set (denominator) | seedling | mature_v1 | mature_v2 |
|---|---|---|---|---|
| Output: absent | All resolved combinations | 1,308/2,682<br>48.8% [46.9, 50.7] | 1,811/2,372<br>76.3% [74.6, 78.1] | 2,479/3,450<br>71.9% [70.3, 73.4] |
| Output: not assessable | All resolved combinations | 100/2,682<br>3.7% [3.0, 4.5] | 75/2,372<br>3.2% [2.5, 4.0] | 8/3,450<br>0.2% [0.1, 0.5] |
| False absence | Reference present | 76/902<br>8.4% [6.7, 10.4] | 80/315<br>25.4% [20.7, 30.6] | 67/614<br>10.9% [8.6, 13.7] |
| Not assessable on an assessable reference | Reference present or absent | 12/2,357<br>0.5% [0.3, 0.9] | 72/2,348<br>3.1% [2.4, 3.9] | 3/3,405<br>0.1% [0.0, 0.3] |
| Not assessable matches reference | Reference not assessable | 88/325<br>27.1% [22.3, 32.3] | 3/24<br>12.5% [2.7, 32.4] | 5/45<br>11.1% [3.7, 24.1] |
| False positive: all eligible traits | Reference absent | 215/1,455<br>14.8% [13.0, 16.7] | 237/2,033<br>11.7% [10.3, 13.1] | 376/2,791<br>13.5% [12.2, 14.8] |
| False positive: structure-trait subset | Reference absent; subset only | 3/496<br>0.6% [0.1, 1.8] | 35/436<br>8.0% [5.7, 11.0] | 3/556<br>0.5% [0.1, 1.6] |

- Each result is n/N followed by the percentage and its exact 95% CI. Unless marked as a subset, results cover the 10, 22, and 18 traits permitting absent in seedling, mature_v1, and mature_v2. The fixed structure subset contains 2, 4, and 3 traits, respectively.
- Absent means biological absence in an assessable sample. Not assessable means insufficient visual evidence. A false positive is a present pxGPT descriptor with an absent reference; pxGPT not assessable outputs remain in that denominator. A false absence is an absent pxGPT descriptor with a present reference.
- These are sample-trait rates, not plant-category detection rates. Plant-category detection is reported in Table S6.

## Supplementary Table S6. Detection of plant structures and recorded errors.

### A. Plant-level detection

| Dataset | Structure category | Detected / positive | Sensitivity [95% CI] | Correct negative / negative | Specificity [95% CI] |
|---|---|---|---|---|---|
| mature_v1 | Head or curd | 1/1 | 1.000 [0.025, 1.000] | 81/105 | 0.771 [0.679, 0.848] |
| mature_v1 | Reproductive | 2/2 | 1.000 [0.158, 1.000] | 99/110 | 0.900 [0.828, 0.949] |
| mature_v1 | Silique | 1/1 | 1.000 [0.025, 1.000] | 109/109 | 1.000 [0.967, 1.000] |
| mature_v2 | Head or curd | 3/8 | 0.375 [0.085, 0.755] | 179/181 | 0.989 [0.961, 0.999] |
| mature_v2 | Broccoli/cauliflower subset | 1/5 | 0.200 [0.005, 0.716] | 183/184 | 0.995 [0.970, 1.000] |
| mature_v2 | Reproductive | 7/11 | 0.636 [0.308, 0.891] | 182/182 | 1.000 [0.980, 1.000] |
| mature_v2 | Storage organ | 9/9 | 1.000 [0.664, 1.000] | 185/186 | 0.995 [0.970, 1.000] |

- Each observation is one plant-category combination. Reference-not-assessable cases are excluded. Sensitivity and specificity have exact binomial intervals. Small positive counts limit precision.
- Categories overlap within plants. The broccoli/cauliflower subset is contained in the head-or-curd category. Counts must not be summed across categories. The positive records represent 3 distinct mature_v1 plants and 25 mature_v2 plants.
- The ten error records (Table S6, panel B) comprise nine category-detection failures and one storage-organ identity error; the latter remains a detected storage organ.

**Supplementary Table S6 (continued). B. Recorded structure errors and condensed model-reported visual evidence.**

All ten records are from mature_v2 and concern eight cultivar IDs. Head/curd and bolting rows represent nine category-detection failures; the storage-organ row is an identity error.

| Cultivar ID | Trait/category | Human reference | pxGPT output | Condensed visual evidence | Description category |
|---|---|---|---|---|---|
| s0024 | Head/curd | broccoli head | absent | Central immature buds described; head criteria not met.† | Stricter criterion |
| s0170 | Head/curd | broccoli head | absent | No head, curd, floral structure or buds reported. | Not visible |
| s0172 | Head/curd | broccoli head | flowering raceme | Terminal buds on an elongated stem interpreted as a flowering raceme. | Different identity |
| s0276 | Head/curd | cauliflower curd | absent | Open foliage reported; no head, curd or floral structures. | Not visible |
| s0334 | Head/curd | leafy head | absent | Inner-leaf cupping described, but leaves judged insufficiently folded. | Stricter criterion |
| s0024 | Bolting | 1 | 0 | Central flower buds described without stem elongation.† | Stricter criterion |
| s0030 | Bolting | 1 | 0 | No floral stem, buds or elongation reported. | Not visible |
| s0168 | Bolting | 1 | 0 | Central structure identified as a young leaf; no floral stem or buds. | Not visible |
| s0170 | Bolting | 1 | 0 | Elongated stem described without visible flower buds.† | Stricter criterion |
| s0268 | Storage organ | turnip swollen hypocotyl | kohlrabi swollen stem | Swollen base interpreted as a kohlrabi-type stem. | Different identity |

- Head/curd refers to reproductive_or_head_structure; bolting refers to bolting_status (original codes 1 and 0); storage organ refers to storage_organ. Descriptor underscores are replaced by spaces for readability. For s0268, the reference descriptor had a 2–1–1 plurality among four annotations; a storage organ was still detected by pxGPT.
- † The supplied description ends with an ellipsis; the complete original response is unavailable. Evidence text is condensed from the available model-generated descriptions. Description categories comprise four stricter-criterion records, four not-visible records and two different-identity records. They summarize the reported text and do not establish why an error occurred.

## Supplementary Table S7. Step 3 comparisons among models.

| Dataset | Model | Annotated samples | Mean accuracy | Difference [95% CI] | Empty values / required fields |
|---|---|---|---|---|---|
| seedling | Claude Sonnet 5 | 284 | 0.619 | Reference | 91/11,928 |
| seedling | Gemma 4 26b | 284 | 0.640 | 0.021 [0.009, 0.032] | 68/11,928 |
| mature_v1 | Claude Sonnet 5 | 112 | 0.727 | Reference | 0/6,958 |
| mature_v1 | GPT 5.6 Luna | 112 | 0.736 | 0.009 [-0.003, 0.022] | 2/6,958 |
| mature_v1 | Gemma 4 26b | 112 | 0.711 | -0.016 [-0.028, -0.004] | 12/6,958 |
| mature_v2 | Claude Sonnet 5 | 203 | 0.761 | Reference | 2/13,850 |
| mature_v2 | Gemma 4 26b | 203 | 0.732 | -0.029 [-0.038, -0.020] | 106/13,850 |

- Accuracy gives each qualitative trait equal weight and uses resolved reference combinations: 9,946 seedling, 4,743 mature_v1, and 7,828 mature_v2. Difference = comparison model minus Claude Sonnet 5; intervals use 2,000 whole-sample bootstrap resamples.
- Output checks use all processed samples in Table S1, including those without human annotations. Every model returned all required fields with zero qualitative descriptor violations. Empty values are distinct from missing fields and from not assessable descriptors.
- These tests use unchanged Step 3 schemas. They do not establish model equivalence or portability of the complete workflow.

## Supplementary Table S8. Effects of Step 3 components.

| Configuration | Mean accuracy | Difference, pp [95% CI] | Paired t-test P | False-positive rate | Structure sensitivity | Empty quantitative outputs |
|---|---|---|---|---|---|---|
| Full | 0.707 | Reference | Not applicable | 849/3,188<br>0.266 | 783/802<br>0.976 | 66/1,666<br>4.0% |
| Single request | 0.715 | 0.84<br>[-0.06, 1.75] | 0.0712 | 797/3,188<br>0.250 | 780/802<br>0.973 | 4/1,666<br>0.2% |
| No visual-evidence description | 0.680 | -2.68<br>[-3.57, -1.79] | 3.28e-08 | 1,024/3,188<br>0.321 | 789/802<br>0.984 | 0/1,666<br>0.0% |
| No trait definitions | 0.692 | -1.42<br>[-2.52, -0.32] | 0.0125 | 792/3,188<br>0.248 | 781/802<br>0.974 | 62/1,666<br>3.7% |
| Text only | 0.718 | 1.09<br>[-0.25, 2.42] | 0.114 | 283/3,188<br>0.089 | 638/802<br>0.796 | 1,384/1,666<br>83.1% |

- Each configuration was run twice on the same 119 mature_v2 plants with GPT 5.6 Luna. Plant-level accuracy was averaged over runs before paired comparison. pp = percentage points. Tests are exploratory and are not adjusted for multiple comparisons.
- Full: images, nine trait groups, trait/descriptor definitions, and visual-evidence descriptions. Single request: all traits together. No visual-evidence description: omit the rationale field and its instruction. No trait definitions: omit definitions and absent/not assessable rules. Text only: use Step 1 descriptions instead of images.
- Outcome cells show counts summed over two runs, followed by the mean of the two run-specific rates. Each run contains 1,594 reference-absent combinations, 401 reference-present combinations, and 833 expected quantitative combinations. These are sample-trait outputs, not additional independent plants.

## Supplementary Table S9. Sensitivity to reference and exclusion rules.

### A. Reference-label rules

| Dataset | Reference-label rule | Evaluated pairs | Mean accuracy | Mean balanced accuracy | Mean macro-F1 |
|---|---|---|---|---|---|
| seedling | Primary: half to even | 9,946 | 0.6190 | 0.5475 | 0.5010 |
| seedling | Exclude rounded-median combinations | 8,260 | 0.6360 | 0.5577 | 0.5180 |
| seedling | Half up | 9,946 | 0.6241 | 0.5472 | 0.5106 |
| seedling | Half down | 9,946 | 0.6009 | 0.5435 | 0.4940 |
| mature_v1 | Primary: half to even | 4,743 | 0.7266 | 0.6114 | 0.5359 |
| mature_v1 | Exclude rounded-median combinations | 4,492 | 0.7359 | 0.6143 | 0.5470 |
| mature_v1 | Half up | 4,743 | 0.7274 | 0.6115 | 0.5431 |
| mature_v1 | Half down | 4,743 | 0.7247 | 0.6090 | 0.5416 |
| mature_v2 | Primary: half to even | 7,828 | 0.7608 | 0.5915 | 0.5554 |
| mature_v2 | Exclude rounded-median combinations | 7,243 | 0.7709 | 0.6001 | 0.5611 |
| mature_v2 | Half up | 7,828 | 0.7606 | 0.5870 | 0.5555 |
| mature_v2 | Half down | 7,828 | 0.7553 | 0.5931 | 0.5511 |

### B. Exclusion of inapplicable child traits

| Dataset | Evaluated combinations | Mean accuracy |
|---|---|---|
| seedling | 9,343 | 0.610 |
| mature_v1 | 4,524 | 0.728 |
| mature_v2 | 7,423 | 0.746 |

- Each alternative is applied separately to the primary analysis. Panel A gives each qualitative trait equal weight for all three metrics. Rounding alternatives affect ordered reference labels; they do not alter pxGPT outputs. Panel A retains four decimal places from the supplied sensitivity results.
- In panel B, the post hoc dependency analysis applies ten accepted parent-child rules (eight high-confidence and two medium-confidence). It excludes child combinations when the human parent reference meets the rule for inapplicability, which can include absence, no open flower, or roots not visible. It removes 603, 219, and 405 resolved combinations in dataset order and does not replace the primary denominator.
- Both panels compare pxGPT outputs with human reference labels. The maximum changes in the Results refer to panel A and do not include the child-trait exclusions in panel B.

## Supplementary Figures

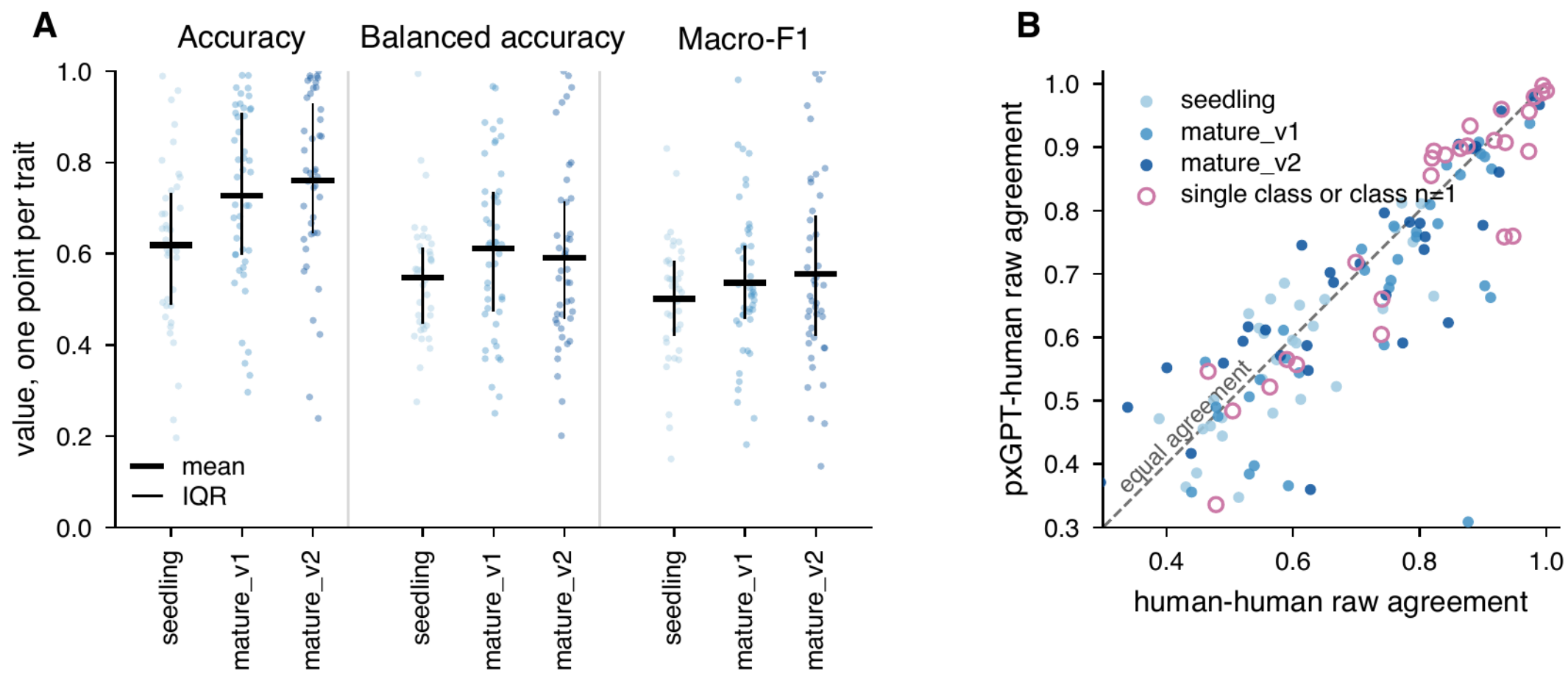


**Supplementary Figure S1. Trait-level validation of qualitative traits.**

(A) Distributions of trait-level accuracy, balanced accuracy, and macro-averaged F1 relative to human reference labels. Individual trait estimates and a summary marker are shown for each dataset. (B) Raw pxGPT-human agreement plotted against human-human agreement for the same traits and sample-trait combinations evaluated by multiple annotators. The dashed line indicates equal agreement. Traits with only one reference class or a reference class represented by a single sample are marked to indicate limitations in assessing class discrimination or estimating uncertainty under repeated sampling. Per-trait accuracy and 95% confidence intervals are reported in Table S2, panels B–D. Dataset-level raw agreement is reported in Table S3. Table S2, panels E–G, summarizes the score distributions and reference-class support.

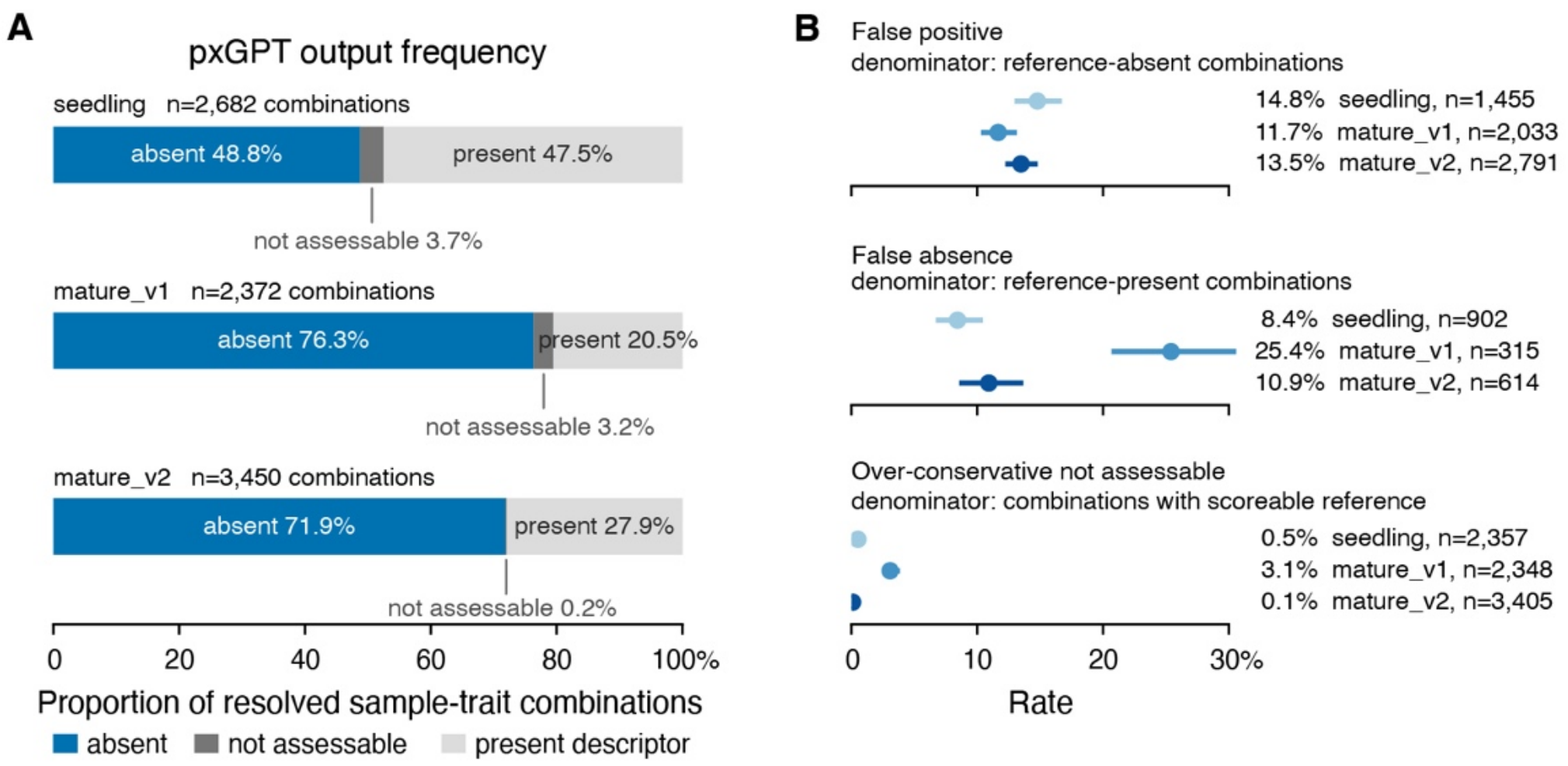


**Supplementary Figure S2. Outcomes for traits with an explicit absent descriptor.**

Panels distinguish pxGPT output frequencies from errors determined using human reference labels. Absent and not assessable are analyzed separately. Rates of false positives, false absence, and overly conservative not assessable outputs use the denominators defined in Supplementary Methods section 6 and listed in Table S5.

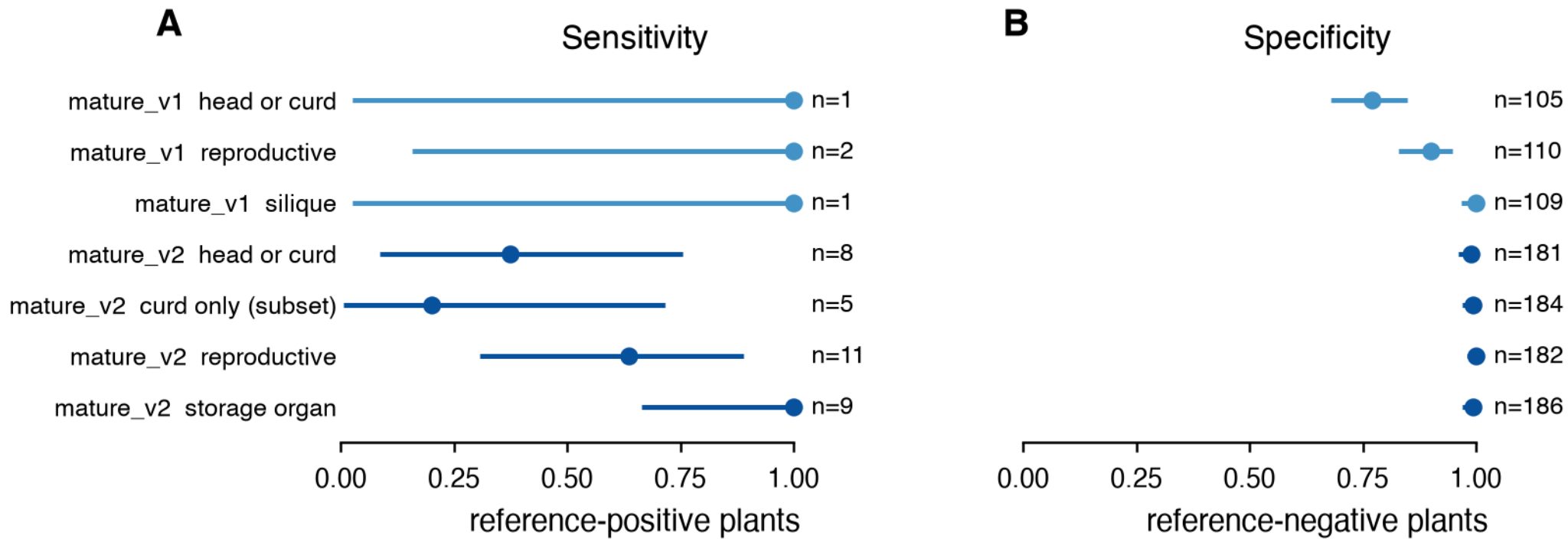


**Supplementary Figure S3. Detection of reproductive structures, head or curd structures, and swollen storage organs by dataset.**

Points show sensitivity or specificity, bars indicate 95% Clopper-Pearson confidence intervals, and labels report the numbers of reference-positive or reference-negative plants, respectively. Sensitivity estimates for mature_v1 are based on only one or two reference-positive plants. The curd-only row for mature_v2 represents the broccoli/cauliflower subset of the head-or-curd category in Table S6. Individual plants may be positive in more than one category; therefore, counts should not be summed across categories.

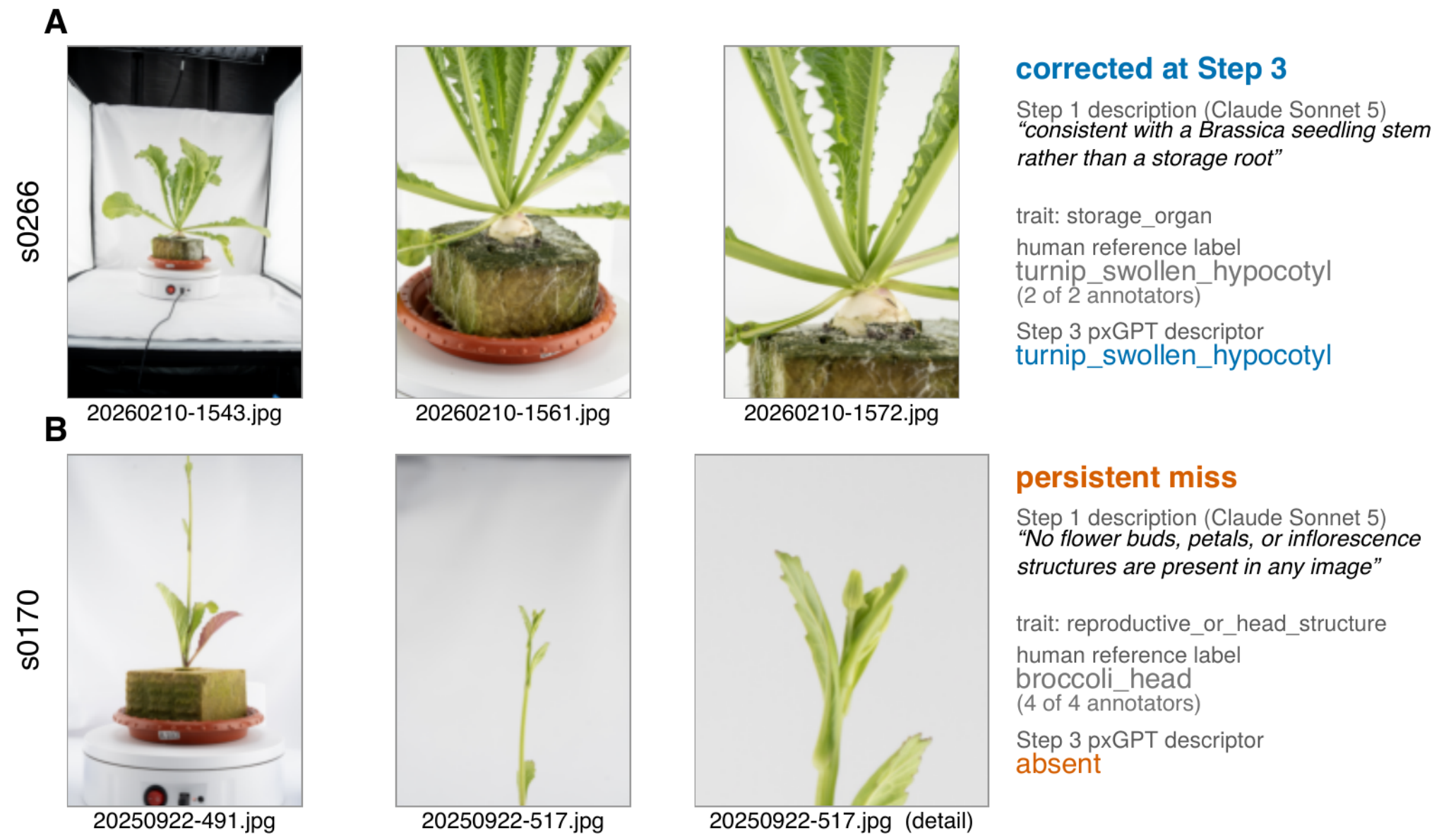


**Supplementary Figure S4. Examples of how schema-constrained extraction can differ from free-text description.**

The cases were selected from a predefined list of mismatches between Step 1 descriptions and the first author's original image review. (A) s0266: Step 1 described a pale, thickened hypocotyl but did not identify a swollen storage organ; Step 3 returned turnip_swollen_hypocotyl, matching both independent annotators. (B) s0170: Step 1 and Step 3 both missed the reproductive stem and terminal buds recorded by four independent annotators. All available views were analyzed for scoring. These cases illustrate one corrected classification and one persistent, missed detection. They are not used to estimate an error rate or establish that schema-constrained extraction systematically corrects errors in free-text descriptions.

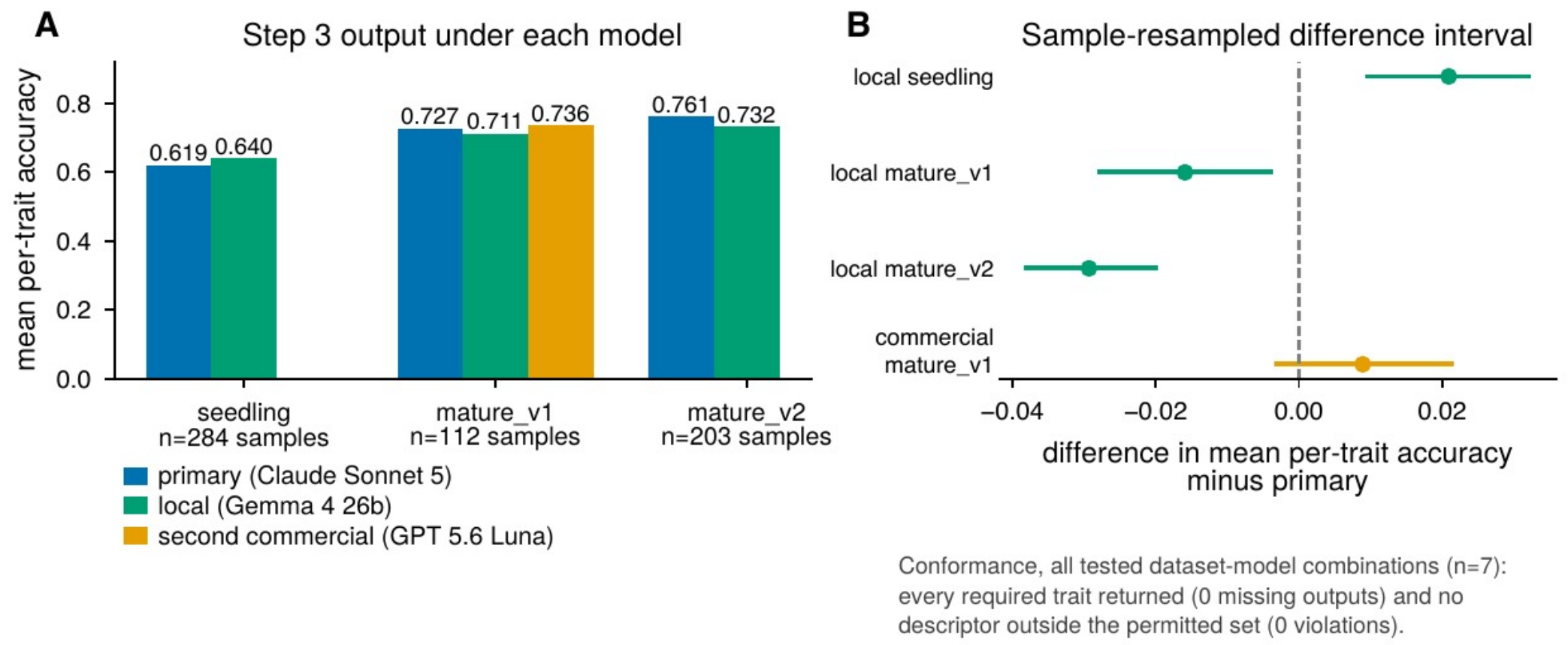


## Supplementary Figure S5. Portability of Step 3 across models.

For each dataset, all models used the same curated schema and human reference labels. The commercial-model comparison covers mature_v1, whereas the local Gemma 4 26b comparison covers all three datasets. (A) Bars show mean accuracy across qualitative traits. (B) Points show differences in mean accuracy between each comparison model and the primary model (comparison minus primary); horizontal bars show 95% confidence intervals obtained by resampling whole samples (Table S7). The figure evaluates the portability of schema-constrained extraction in Step 3, not full-workflow reproducibility or model equivalence.

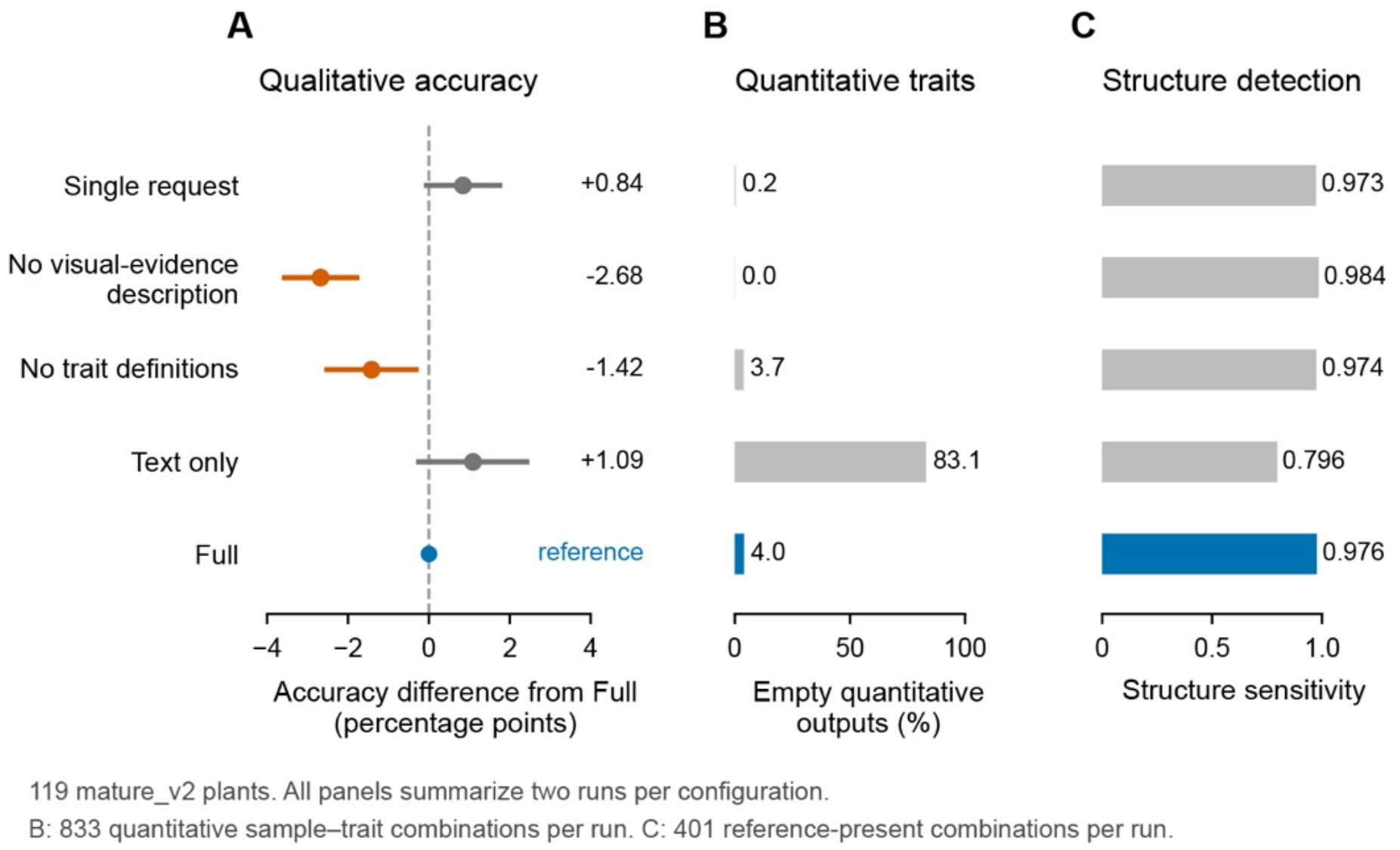


## Supplementary Figure S6. Component ablation analysis in 119 mature_v2 plants.

Each configuration was run twice using GPT 5.6 Luna. (A) Points show mean paired differences in accuracy for qualitative traits relative to the full configuration, expressed in percentage points. For example, accuracy was 0.6798 for the “No visual-evidence description” configuration and 0.7066 for the full configuration, giving a difference of -0.0268, equivalent to –2.68 percentage points. Accuracy was averaged across the two runs within each plant before calculating paired differences. Bars indicate 95% confidence intervals for the mean paired difference across 119 plants. (B) Frequency of empty quantitative outputs across 833 sample-trait combinations per run for all five configurations. (C) Sensitivity across the 401 sample-trait combinations per run for which the human reference indicated structure presence. Panels B and C show the mean of the two run-specific rates. The “No visual-evidence description” configuration removes the visual-evidence description (stored in the rationale field) and the instruction to generate it. The “No trait definitions” configuration removes trait and descriptor definitions and the rules for absent and not assessable (Table S8). Tests were exploratory, with no adjustment for multiple comparisons.
